# Encapsulation Enhances the Quantum Coherence of a Solid‐State Molecular Spin Qubit

**DOI:** 10.1002/anie.202510603

**Published:** 2025-09-01

**Authors:** Abinash Swain, Leoní A. Barrios, Yulia Nelyubina, Simon J. Teat, Olivier Roubeau, Valentin Novikov, Guillem Aromí

**Affiliations:** ^1^ Departament de Química Inorgànica i Orgànica Universitat de Barcelona Diagonal 645 Barcelona 08028 Spain; ^2^ Department Institute of Nanoscience and Nanotechnology of the University of Barcelona (IN2UB) University of Barcelona Barcelona Spain; ^3^ Nesmeyanov Institute of Organoelement Compounds Russian Academy of Sciences Moscow 119334 Russia; ^4^ Advanced Light Source Berkeley Laboratory 1 Cyclotron Road Berkeley CA 94720 USA; ^5^ Instituto de Nanociencia y Materiales de Aragón (INMA) CSIC and Universidad de Zaragoza Plaza San Francisco s/n Zaragoza 50009 Spain

**Keywords:** Molecular spin‐qubit, Quantum coherence, Rabi oscillations, Supramolecular helicates, Time‐resolved EPR

## Abstract

Spins within molecules benefit from the atomistic control of synthetic chemistry for the realization of qubits. One advantage is that the quantum superpositions of the spin states encoding the qubit can be coherently manipulated using electromagnetic radiation. The main challenge is the fragility of these superpositions when qubits are to partake of solid‐state devices. We address this issue with a supramolecular approach for protecting molecular spin qubits against decoherence. The molecular qubit [Cr(ox)_3_]^3−^ has been encapsulated inside the diamagnetic triple‐stranded helicate [Zn_2_L_3_]^4+^ (L is a *bis*‐pyrazolylpyridine ligand). The quantum coherence of the protected qubit is then analyzed with pulsed EPR spectroscopy and compared with the unprotected qubit, both in solution and in the solid state. Crucially, the spin–spin relaxation in the solid state has been examined within diamagnetic crystal lattices of the isostructural ([Al(ox)_3_]@[Zn_2_L_3_])^+^ or [Al(ox)_3_]^3‐^ assemblies, respectively, doped with the Cr^3+^ qubit in two different (<10%) concentrations. The study unveils a surprising increase of the phase memory time of the qubit upon encapsulation only in the solid. Spin‐lattice relaxation times also exhibit a significant enhancement, as established from inversion recovery pulse sequences and from slow relaxation of the magnetization of the protected qubit, not featured by the free qubit.

## Graphical abstract

A purely synthetic supramolecular chemistry approach enables the protection of the spin‐based qubit [Cr(ox)_3_]^3−^ from external sources of decoherence in the solid state, leading to the enhancement of the phase memory and the spin lattice relaxation time, thus validating this chemical engineering strategy for qubit implementation.

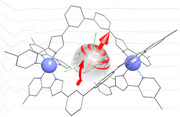

The ongoing second quantum revolution is set to drastically advance current technologies and profoundly reshape our way of life. This transformation is driven by the ability to harness quantum properties through increasingly precise control of matter at the nanoscale.^[^ ^1^ ^]^ One of the most anticipated breakthroughs is quantum computing (QC), which leverages fundamental principles of quantum mechanics (such as superposition and entanglement) to process information via quantum algorithms.^[^ ^2^ ^]^ To realize this potential, quantum computing requires technologies that meet critical requirements, including scalability and quantum error correction. Achieving these capabilities necessitates wiring up a sufficiently large number of identical qubits, a challenge that continues to constrain even the most advanced implementations.^[^ ^3^, ^4^ ^]^ A promising emerging alternative involves using electronic or nuclear spins within molecules to create qubits and quantum gates.^[^ ^5^, ^6^, ^7^, ^8^ ^]^ This approach enables the development of multiqubit architectures with atomic‐level control over topology, interqubit interactions, and the surrounding environment, offering unmatched reproducibility. Molecular quantum processors capable of implementing quantum error correction with a small number of qubits have already been proposed.^[^ ^3^, ^9^ ^]^ Additionally, chemical engineering has helped significantly to increase the quantum coherence of electronic spins of molecules.^[^ ^10^, ^11^, ^12^, ^13^, ^14^, ^15^, ^16^, ^17^, ^18^, ^19^ ^]^ At this stage, the challenge is to preserve the quantum superpositions of qubits when they become part of hybrid solid‐state devices for their implementation.^[^ ^20^, ^21^, ^22^ ^]^ One promising approach is their encapsulation into cages, protecting them from sources of decoherence while being transferred to the appropriate locations. Using fullerenes to host qubits is a promising strategy to achieve this goal but limits enormously the ability to chemically tune the nature and environment of the qubits.^[^ ^23^, ^24^, ^25^, ^26^, ^27^ ^]^

We employ here a purely synthetic chemistry approach to protect spin qubits in the solid state by encapsulating them within a suited supramolecular host. As a qubit realization, we selected the Cr(III) center in the complex [Cr(ox)_3_]^3−^ (ox = dianion of oxalic acid), which has been shown to coherently encode qubits through any of the transitions within its axially anisotropic *S* = 3/2 spin state.^[^ ^28^, ^29^ ^]^ We show that this complex can be adequately positioned inside the cavity of a diamagnetic [Zn_2_L_3_]^4+^ supramolecular triple‐stranded helicate (L being a ligand formed by a biphenyl group separating two pyrazolylpyridine coordination pockets). The protected qubit is obtained by reaction of K_3_[Cr(ox)_3_] with a solution of ZnCl_2_ and L in the presence of 18‐crown‐6 as crystals of the ionic assembly [Cr(ox)_3_]@[Zn_2_L_3_]Cl (**1**). Its single‐crystal X‐ray diffraction (SCXRD) analysis (Figures 1, S1, and S2) reveals that the octahedral qubit is suited to fit the cavity of the host with the assistance of six [O⋯H─N] hydrogen bonds between the oxalate donor O‐atoms and the H‐atoms riding the pyrazolyl rings of the L ligands. The chirality of the ZnN_6_ octahedral centers of the host is correlated with that of the central [Cr(ox)_3_]^3−^ moiety, in turn defining the handedness of the helical architecture. Both enantiomers of the ensemble are present in the monoclinic lattice of **1** (*C*2/c space group, see Tables S1–S3). This lattice contains four formula units of the compound, maintaining the Cr(III) ions separated 12.318 Å apart or more.

**Figure 1:**
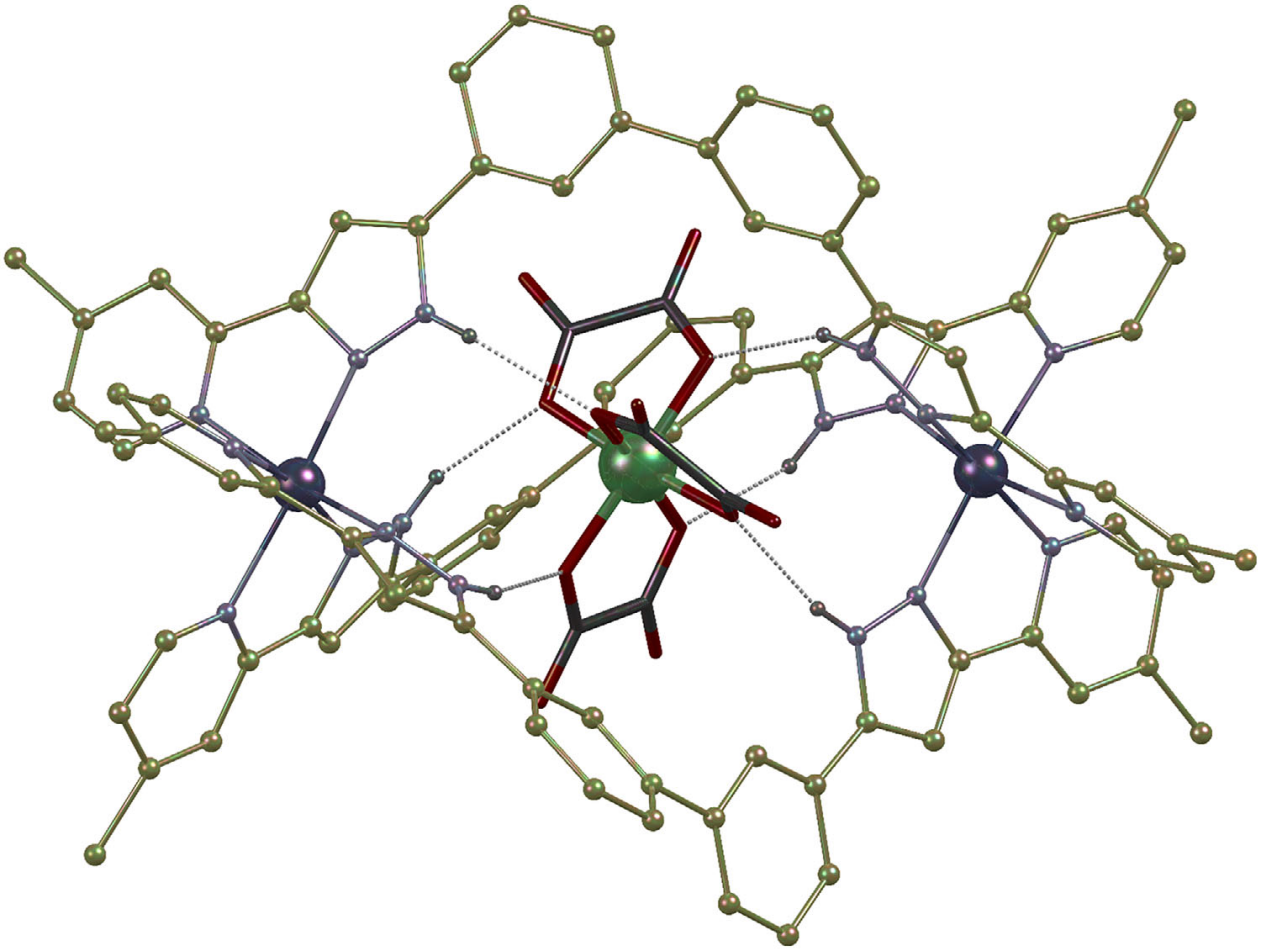
Molecular structure of the cationic assembly ([Cr(ox)_3_]@[Zn_2_L_3_])^+^ in **1**. The [Zn_2_L_3_]^4+^ host is represented in ball‐and‐stick style (where C, yellow balls; Zn, large deep‐purple balls; N, medium purple balls; and H, small purple balls). The encapsulated species is in a capped stick style except for the Cr atom (C, grey; O, red; and Cr, green). Hydrogen atoms but these from N─H groups are omitted. Hydrogen bonds are shown as black dotted lines.

The full ([Cr(ox)_3_]@[Zn_2_L_3_])^+^ assembly was observed by MALDI‐TOF mass spectrometry (MS) thus corroborating its integrity in H_2_O:MeOH solution (Figure S3), while the magnetic structure of the encapsulated [Cr(ox)_3_]^3−^ qubit was confirmed by CW X‐band EPR in DMF solution (Figure S4).

For the proposed modular approach to qubit protection, the quantum dynamic behavior in the solid state is particularly relevant. To investigate these properties, it was essential to isolate the encapsulated qubits within a matrix that maintains a large separation between them. To do so, a diamagnetic analogue of the crystal lattice of **1** —the compound [Al(ox)_3_]@[Zn_2_L_3_]Cl (**2**)— was synthesized using Al instead of Cr. It is isostructural with **1** (Figures S5 and S6, Tables S1–S3) and exhibits analogous MS properties (Figure S7). This structural correspondence suggests the possibility of doping the lattice of **2** with dispersed ([Cr(ox)_3_]@[Zn_2_L_3_])⁺ supramolecular cations to form a solid solution. This doping was successfully achieved at two different concentrations, resulting in lattices with ICP‐determined molar Al:Cr ratios of 0.91:0.09 (**3**) and 0.97:0.03 (**4**), respectively, obtained using mixtures of K_3_[M(ox)_3_] (M = Al and Cr) in the respective stoichiometric amounts. The SCXRD structure of **4** (Figure S8 and Tables S1–S3) is analogous to that of **1** and **2** and agrees with the ICP results (see Supporting Information). The MS of the microcrystalline molecular alloys unveils both versions of ([M(ox)_3_]@[Zn_2_L_3_])⁺ (M = Al and Cr) in the appropriate proportions (Figures S9 and S10). The isostructural nature of **1**, **2**, **3**, and **4** in the bulk was confirmed by powder X‐ray diffraction (PXRD) at room temperature (Figure S11) as well as IR spectroscopy (Figure S12).

The magnetic properties of the [Cr(ox)_3_]^3−^ qubit in the lattices of **1**, **3**, and **4** were studied by magnetometry and X‐band EPR spectroscopy. For all these measurements, the solids used did certainly not retain the same solvate molecules as those observed by SCXRD. The composition was expected to coincide with that determined by microanalysis (see Supporting Information), thus leading to a slightly modified crystal lattice. The CW EPR spectra exhibit the typical features of an *S* = 3/2 system with slight anisotropy, which is satisfactorily simulated with *g*_x_ = 1.86(7), *g*_y_ = 1.90(9), *g*_z_ = 1.94(7), *D* = 0.660(6) cm^−1^, and *E* = 0.030(0) cm^−1^. No significant variations are observed among the spectra of undiluted and diluted solid‐state samples or with liquid‐phase DMF solution measurements (Figure 2, of **4**, where the complex acts as dopant in 3%; S4, of **1** dissolved in DMF; Figure S14, of solid **1**, with 100% of the guest being the Cr(III) complex), thus confirming the persistence of the encapsulated qubit's magnetic parameters across different environments. The variable field and temperature magnetization data of **1** are consistent with a paramagnetic species with *S* = 3/2 and *g *= 2.17 and are reproduced using the same spin Hamiltonian parameters as for the CW EPR spectra (Figure S13). Compound **3** exhibits the same behavior if an Al:Cr ratio of 0.94:0.06 is considered, in reasonable agreement with ICP results (vide supra).

**Figure 2:**
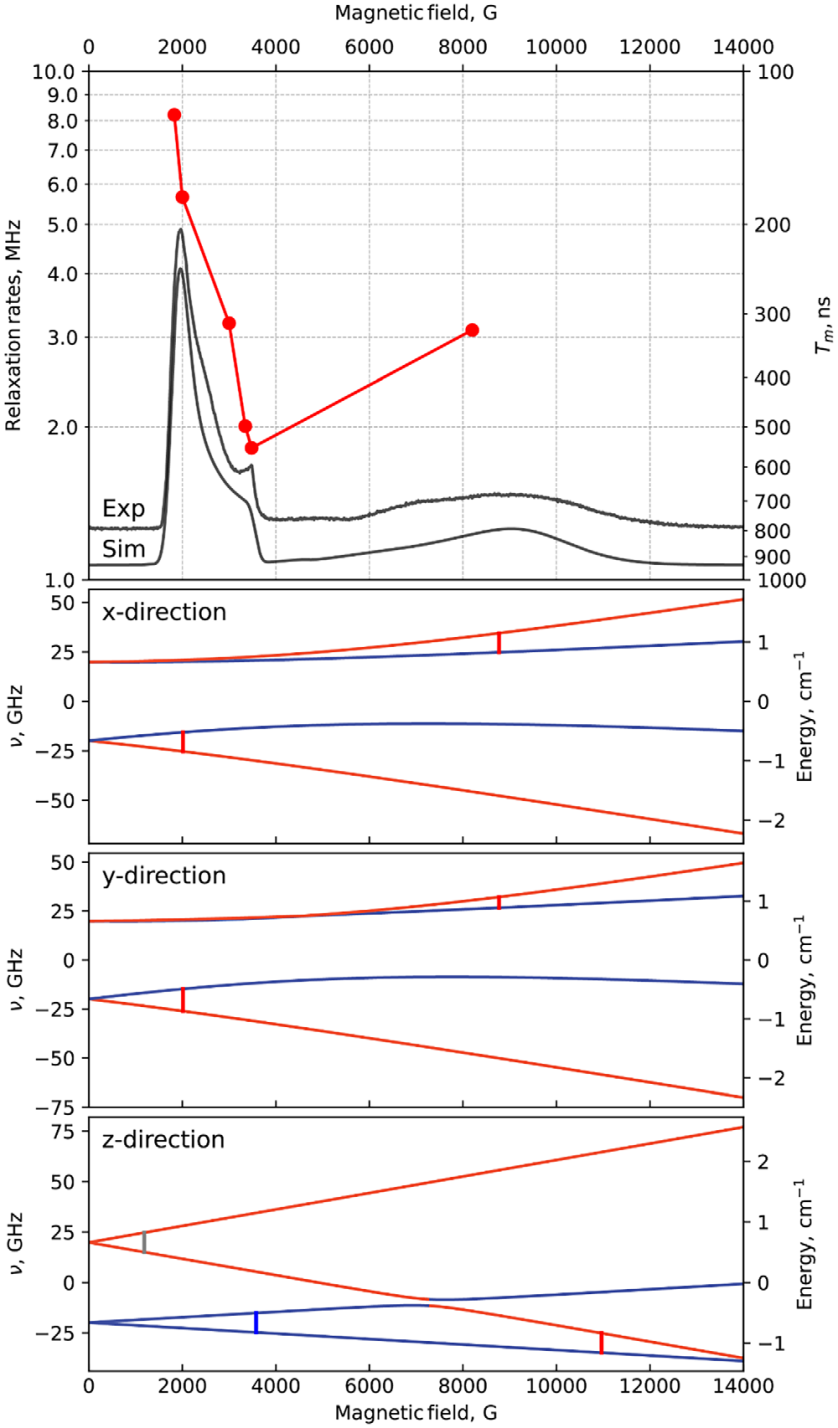
Top panel: Pulsed EPR data of a polycrystalline sample of **4**, without grinding, at 10 K (X‐band): field‐dependence of the phase memory time and corresponding relaxation rates (●, connecting lines are a guide to the eye), plotted on the same magnetic field axis as the experimental and simulated echo detected field sweep (EDFS) EPR spectra of **4**. Lower panels: Energy level diagrams for different orientations of the magnetic field, corresponding to the simulated spectrum with *g*_x_ = 1.86(7), *g*_y_ = 1.90(9), *g*_z_ = 1.94(7), *D* = 0.660(6) cm^−1^, and *E* = 0.030(0) cm^−1^. The colors of the levels correspond to the predominant contribution of the *m*_s_ = ±1/2 (blue) or *m*_s_ = ± 3/2 (red) states. Inter‐Kramers transitions within the *m*_s_ = ±1/2 states are shown in blue, transitions between states with different |*m*_s_| values in red, forbidden transitions in gray.

The dynamic magnetic properties were first investigated with frequency‐dependent ac susceptibility on the concentrated solid compound **1**. At 2 K, the application of a dc field as low as 0.025 T induces the rise of an out‐of‐phase component of the magnetic susceptibility, *χ″*, indicating slow relaxation of the magnetization (Figures S15–S17). This unique behavior for the Cr(III) ion was originally reported by us in the [Cr(ox)_3_]^3−^ complex upon encapsulation by a ferrous [Fe_2_L_3_]^4+^ cage.^[^ ^30^ ^]^ In **1**, a slower relaxation mode of the ([Cr(ox)_3_]@[Zn_2_L_3_])^+^ species emerges for applied dc fields above 0.2 T. The characteristic relaxation times, *τ*, for the main, faster mode reach up to 10 ms for *B* = 2000 G. Temperature‐dependent measurements across a temperature range of 2–7 K were also performed at this applied dc field. The thermal dependence of *τ* follows a power‐law with an exponent close to −2 (*τ* ∝ *T* ^−1.94^, Figure S17), consistent with a direct relaxation process dominated by a phonon bottleneck. The latter may be due to a relaxation pathway mediated exclusively by the molecular vibrations of the encapsulated qubit while isolated from those of the lattice, in sharp contrast to the absence of slow relaxation of the magnetization observed for K_3_[Cr(ox)_3_], even upon the application of an external dc field.^[^ ^30^ ^]^ We recently suggested the emergence of such sublattice of vibrations for the relaxation in guest@[Co_2_] helicates.^[^ ^31^ ^]^

The spin‐lattice relaxation times *T*_1_ of [Cr(ox)_3_]^3−^ in **3** and **4** were also determined using pulsed EPR at various temperatures (4–25 K) and *B* = 3480 G through inversion recovery experiments (Figures S18 and S19). The resulting *T*_1_ values, determined by fitting the echo intensity curves to stretched exponential functions, are consistent with the relaxation times *τ* obtained from ac magnetic susceptibility studies on **1** (Figure 3a), further supporting the presence of a dominant phonon bottleneck. This coincidence is surprising considering that both methods of determination are very different, one based on a thermodynamic bulk measurement and the other being spectroscopic, in addition to the fact that the magnetic measurements are performed on a pure compound whereas the pulsed EPR determinations are performed on diluted samples.

**Figure 3:**
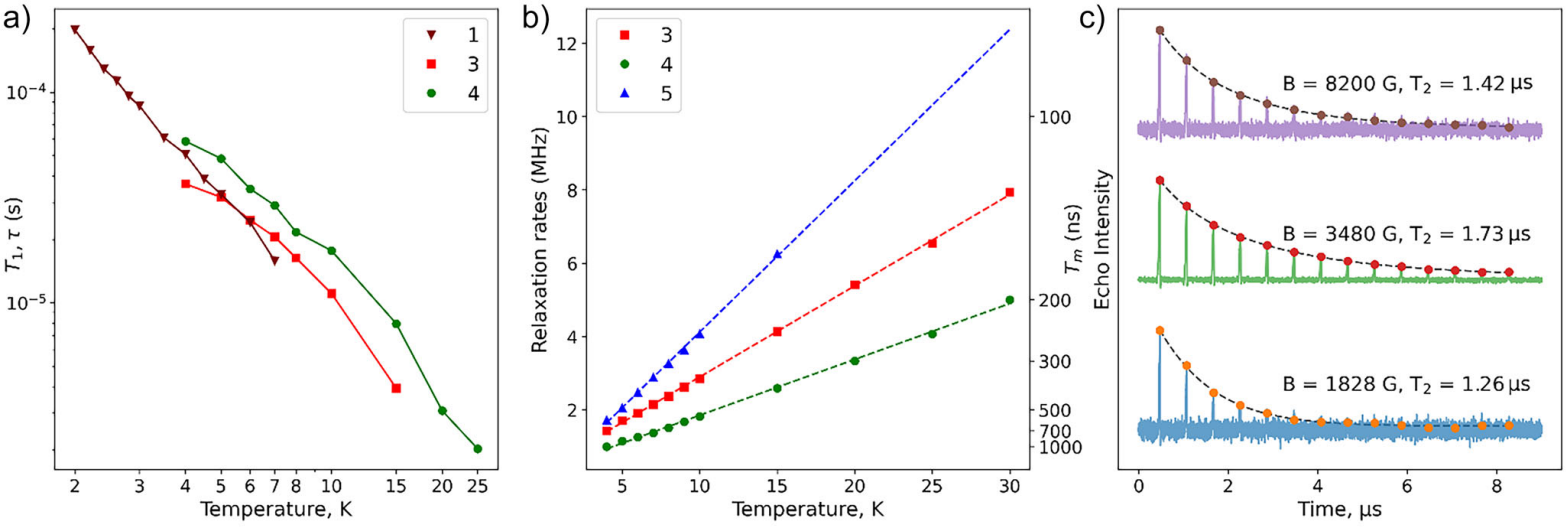
Relaxation data obtained by pulsed EPR and magnetic susceptibility measurements: a) Temperature‐dependence of spin–lattice relaxation times for ([Cr(ox)_3_]@[Zn_2_L_3_])^+^ at different Cr(III) concentrations in the solid‐state solutions. Plots are from Cr(III) contents of 100% (**1**, by *ac*‐magnetometry), 9% and 3% (**3** and **4**, by EPR), lines are to guide the eye. b) Temperature‐dependence of decoherence rates for **3**, **4**, and **5**; c) Results of CPMG for **4**, showing the transient responses at 1828, 3480, and 8200 G at 4 K, with amplitudes estimated by integrating each echo and fitting with a stretched exponential.

The effect of the encapsulation on the solid‐state quantum coherence of the [Cr(ox)_3_]^3−^ qubit was then analyzed by pulsed EPR on **3** and **4**. In both cases, the EDFS spectra are identical to those observed in CW measurements both for the magnetically concentrated solid samples and DMF solutions (Figures S20 and S21) and are simulated using the same spin Hamiltonian parameters (see above). The phase memory time of **4** at different magnetic fields was evaluated by fitting the Hahn echo decay curves to stretched exponentials (Figure S22). As relaxation times are affected by the nature of the corresponding EPR transitions,^[^ ^29^ ^]^ we analyzed the Zeeman diagrams associated with them (Figure 2). The contributions of the different spin manifolds to EPR transitions have a clear impact on their associated *T*_m_. The longest relaxation times (i.e., slowest relaxation rates) are observed at magnetic fields corresponding to transitions among the *m*_s_ = ±1/2 states along the z‐axis. In contrast, inter‐Kramers transitions result in significantly shorter *T*_m_ (Figure 2).

The temperature dependence of *T*_m_ in the solid state was investigated for the most coherent transition (at *B* = 3480 G). As expected, the magnetic relaxation in the sample with 3% of Cr is slower than in that with 9%, both showing strong temperature dependence (e.g., 1 µs versus 0.7 µs at 4 K; Figures S23 and S24). In all cases, *T*_m_ remains smaller than *T*_1_ by more than one order of magnitude; therefore, the spin‐lattice relaxation does not limit quantum coherence in these systems.

Note that the phase memory times at all temperatures were significantly longer than those of the free [Cr(ox)_3_]^3−^ species, even when the latter was measured within the crystal lattice of K_3_[Al(ox)_3_] doped with ∼1% of [Cr(ox)_3_]^3−^ (**5**). Thus, *T*_m_ in **5** is shorter than in **3**, which contains 9% of paramagnetic ions, even though a simple model based on the average Cr⋯Cr distance (see Supporting Information: Discussion) predicted much stronger dipole–dipole interactions in **3**. Such behavior indicates that dipolar interactions are not the main cause of decoherence in this system. A more concentrated version of compound **5**, with 7% Cr (**6**), was also prepared (see Supporting Information) but is not discussed here.

Further insight into the mechanism of spin relaxation comes from a combined analysis of the field and temperature dependences of *T*_m_. For samples **3**–**5**, the spin–spin relaxation rates (1/*T*_m_) scale linearly with temperature (Figure 3b), strongly suggesting a phonon‐driven decoherence.^[^ ^32^ ^]^ Although phonon‐induced processes are typically associated with spin‐lattice relaxation^[^ ^33^ ^]^ and affect *T*_1_, they can also reduce *T*_m_ by modulating terms in the spin Hamiltonian, thereby shifting the resonance frequency and causing spectral diffusion and accelerated decoherence. The field‐dependence of *T*_m_, which shows faster decoherence at positions corresponding to inter‐Kramers transitions, supports this interpretation (Figure 2). These transitions experience stronger spin–orbit coupling and are more sensitive to phonon‐induced fluctuations of the zero‐field splitting than transitions within the *m*_s_ = ±½ doublet, leading to shorter coherence times. Along these lines, applying a Carr–Purcell–Meiboom–Gill (CPMG) sequence^[^ ^34^ ^]^ to sample **4** increased the phase memory time and markedly reduced its field dependence (Figure 3c). The narrower *T*_m_ range under CPMG (1.26–1.70 µs) compared to its nearly five‐fold variation with only Hahn echo sequences (Figure 2) suggests that phonon‐driven spectral diffusion dominates decoherence in this regime.

From the above analysis, it follows that the increased coherence time in the solid state observed for ([Cr(ox)_3_]@[Zn_2_L_3_])^+^ following the encapsulation of the [Cr(ox)_3_]^3−^ qubit into the cavity of the supramolecular helicate can be attributed to the reduced phonon contribution to decoherence. It likely arises from significant differences in the phonon environments of the two hosts. In the inorganic K_3_[Al(ox)_3_] lattice, strongly coupled phonon modes efficiently interact with the electronic states of Cr(III) ions. In contrast, encapsulating individual [Cr(ox)_3_]^3−^ units in a relatively flexible metal–organic cavity leads to lower phonon density, weaker vibrational coupling to the metal center, and greater decoupling of the spin from the rigid lattice.

Further supporting this conclusion, dissolution of free [Cr(ox)_3_]^3−^ leads to a significant increase in *T*_m_ (Figure S25), consistent with the disappearance of strong lattice phonons. On the contrary, dissolving ([Cr(ox)_3_]@[Zn_2_L_3_])^+^ does not improve its relaxation properties. Indeed, a standard Hahn echo pulse sequence of the latter species^[^ ^35^ ^]^ yields a phase memory time of *T*_m_ = 602 ns at 4 K (Figure S26), similar to that derived in the solid state, while the EDFS spectrum (Figure S4) of the solution agrees with its CW counterpart. This indicates that the vibrations involved in the relaxation remain the same as in the solid state and are localized rather than extended, also in agreement with the proposed origin of phonon bottleneck affecting *T*_1_.

Taken together, these results confirm the validity of the encapsulation approach as a strategy for stabilizing quantum superpositions of qubits in the solid state by isolating them from lattice vibrations.

The spin dynamics of [Cr(ox)_3_]^3−^ in sample **4** was further explored via nutation experiments at different magnetic fields. The echo decay as a function of nutation pulse length shows Rabi oscillations (Figures S27–S29), with frequencies, Ω_R_, proportional to microwave field *B*_1_, confirming the possibility to reliably manipulate the qubit and put it into arbitrary superposition states for all the tested fields. The best qubit figure of merit extracted from these experiments is *T*_m_/*T*_G _= 216, where *T*_G_ is the gate time defined as the duration of a π‐pulse (8 ns) at the highest applied microwave power of 3.5 dB at magnetic field of 3480 G.

In conclusion, we have demonstrated that encapsulating the molecular spin‐qubit [Cr(ox)_3_]^3−^ within the diamagnetic triple‐stranded helicate [Zn_2_L_3_]⁴⁺ significantly improves its quantum coherence in the solid state. Through extensive use of pulsed EPR spectroscopy, we observed a notable increase in phase memory time and spin‐lattice relaxation time compared to the unprotected qubit. The encapsulation mitigates decoherence mainly by reducing phonon coupling to the chromium ion; the softer, vibrationally decoupled environment of the supramolecular host lowers the impact of spectral diffusion and extends the phase memory time. More broadly, our findings challenge the prevailing trade‐off between chemical functionality and quantum performance: while synthetic modifications often introduce magnetic noise and compromise coherence, we demonstrate that embedding the qubit within a large supramolecular host can instead enhance it. This opens the door to incorporating switchable units, tunable spin–spin couplings, or surface anchoring groups without sacrificing coherence—a critical step toward functional quantum molecular devices.

## Supporting Information

Synthesis procedure, spectroscopic data,^[^ ^36^ ^]^ and crystallographic details. This material is available free of charge via the Internet at http://XX. The authors have cited additional references within the Supporting Information.^[^ ^37^, ^38^, ^39^, ^40^, ^41^, ^42^, ^43^, ^44^, ^45^, ^46^, ^47^, ^48^ ^]^

## Conflict of Interests

The authors declare no conflict of interest.

## Supporting information



Supporting Information

## Data Availability

The data that support the findings of this study are available from the corresponding author upon reasonable request.
