# Supplementary material for: Encapsulation Enhances the Quantum Coherence of a Solid‐State Molecular Spin Qubit
Source: Angew Chem Int Ed Engl. 2025 Sep 1;64(42):e202510603. doi: 10.1002/anie.202510603 (PMC12518696; doi:10.1002/anie.202510603)
Supplement: Supplementary file 1 — Supporting Information [file ANIE-64-e202510603-s001.pdf]

Encapsulation Enhances the Quantum Coherence of a Solid-State Molecular Spin-Qubit

Abinash Swain,[#,$] Leoní A. Barrios,[#,$] Yulia Nelyubina,[&] Simon Teat, Olivier Roubeau,[§] Valentin Novikov[#,$,]* and Guillem Aromí[#,$,]*

[#] Departament de Química Inorgànica i Orgànica, Universitat de Barcelona, Diagonal 645, 08028 Barcelona, Spain.

[$] Institute of Nanoscience and Nanotechnology of the University of Barcelona (IN2UB), Barcelona, Spain.

[§] Instituto de Nanociencia y Materiales de Aragón (INMA), CSIC and Universidad de Zaragoza, Plaza San Francisco s/n, 50009, Zaragoza, Spain.

[&] Nesmeyanov Institute of Organoelement Compounds, Russian Academy of Sciences, 119334 Moscow, Russia

[†] Advanced Light Source, Berkeley Laboratory, 1 Cyclotron Road, Berkeley, California 94720, USA

Supporting Information

Table of Contents:

1. Synthesis
2. Physical Measurements
3. Single Crystal X-Ray Diffraction
4. EPR spectroscopy
5. Magnetometry
6. Figures
7. Tables

1. Synthesis

Ligand L (L = 3,3'-*bis*(3(4-picolin-2-yl)-1*H*-pyrazol-5-yl)-1,10-biphenyl) was prepared using our previously published procedure.[1] The complex salt $K_3[Cr(ox)_3]·3H_2O$ was bought from Sigma-Aldrich and used without further purifications. The analogous compound $K_3[Al(ox)_3]·4.5H_2O$ was synthesized using a previously reported procedure.[2]
$[Cr(ox)_3]@[Zn_2L_3]Cl$ (1): Ligand L (28.1 mg, 0.06 mmol) in methanol (8 ml) was added dropwise to a stirred solution of $ZnCl_2$ (5.45 mg, 0.04 mmol) in methanol (4 ml) and the solution was stirred for 1 hour and filtered. The filtrate was added to a solution of $K_3[Cr(ox)_3]·3H_2O$ (9.7 mg, 0.02 mmol) and an excess of 18-crown-6 (20 mg, 0.075 mmol) in DMF (4 ml) that had also been stirred for 1 hour and filtered previously. After 4 days, this resulted in the crystallization of complex $[Cr(ox)_3]@[Zn_2L_3]Cl$ (**1**) as purple needle shape that were suitable for single crystal X-ray diffraction. Anal. Calcd (%) for **1**·15H_2O, (found): C, 53.43 (53.11); H, 4.76 (4.16); N, 11.68 (12.00). MS: *m/z* = 1852.2001

---

[1] Capo, N.; Barrios, L. A.; Cardona, J.; Ribas-Ariño, J.; Teat, S. J.; Roubeau, O.; Aromí, G., The template effect of a SiF_6^{2-} guest drives the formation of a heteroleptic Fe(II) coordination helicate. *Chem. Commun.* **2022,** *58*, 10969-10972.
[2] Synthesis of $K_3[Al(Ox)_3]·4.5H_2O$, Pass, G.; Sutcliffe, H. Practical Inorganic Chemistry – 2nd edition. Chapman and Hall: London, 1974, pp. 11.

([Cr(ox)$_3$]@[Zn$_2$L$_3$])$^+$; 1384.0408 ([Cr(ox)$_3$]@[Zn$_2$L$_2$])$^+$; 1294.1509 ([Cr(ox)$_2$]@[Zn$_2$L$_2$] − 2H$^+$)$^+$.

[Al(ox)$_3$]@[Zn$_2$L$_3$]Cl (2): Ligand L (28.1 mg, 0.06 mmol) in methanol (8 ml) was added dropwise to a stirred solution of $ZnCl_2$ (0.04 mmol, 5.45 mg) in methanol (4 ml). The solution was stirred for 1 hour and combined with a solution of K_3[Al(ox)$_3$]·4.5H_2O (9.78 mg, 0.02 mmol) and an excess of 18-crown-6 (20 mg, 0.075 mmol) in DMF (4 ml) that had also been stirred for 1 hour. The mixture was stirred for 15 minutes and filtered. White needle shaped crystals suitable for single crystal X-ray diffraction started to form in 4 days, following vapor diffusion of diethyl ether. Anal. Calcd (%) for **2**·15H_2O, (found): C, 54.05 (53.53); H, 4.82 (3.75); N, 11,82 (11.04). MS: *m/z* = 1827.0632 ([Al(ox)$_3$]@[Zn$_2$L$_3$])$^+$; 1381.8958 ([Al(ox)$_3$]@[Zn$_2$L$_2$] – H$^+$ + Na$^+$)$^+$; 1358.9354 ([Al(ox)$_3$]@[Zn$_2$L$_2$])$^+$; 1266.9711 ([Al(ox)$_2$]@[Zn$_2$L$_2$] − 2H$^+$)$^+$.

([Al(ox)$_3$]$_{0.91}$[Cr(ox)$_3$]$_{0.09}$)@[Zn$_2$L$_3$]Cl (3): The same procedure as for compound **2** was followed, replacing the K_3Al(ox)$_3$ salt with a mixture of K_3[Al(ox)$_3$]·4.5H_2O (8.80 mg, 0.018 mmol) and K_3[Cr(ox)$_3$]·3H_2O (0.97 mg, 0.002 mmol). Metal analysis, ICP (% of M wrt Cr + Al, found): Al, 90.7; Cr, 9.3.

([Al(ox)$_3$]$_{0.97}$[Cr(ox)$_3$]$_{0.03}$)@[Zn$_2$L$_3$]Cl (4): The same procedure as for compound **3** was followed, using the mixture of K_3[Al(ox)$_3$]·4.5H_2O (9.68 mg, 0.0198 mmol) and K_3[Cr(ox)$_3$]·3H_2O (0.09 mg, 0.0002 mmol). Metal analysis, ICP (% of M wrt Cr + Al, found): Al, 96.6; Cr, 3.4.

K_3[Al$_{0.93}$Cr$_{0.07}$(ox)$_3$]: Solid K_3[Cr(ox)$_3$]·3H_2O (0.97 mg, 0.002 mmol) was added to a boiling aquous solution of K_3[Al(ox)$_3$]·4.5H_2O (8.81 mg, 0.018 mmol) in 10 ml of H_2O, and stirring was maintained for 30 minutes. The solution was then kept standing and allowed to cool to room temperature. Upon slow evaporation, large block of crystals starts appearing in seven days. Metal analysis, ICP (% of M wrt Cr + Al, found): Al, 92.8. Cr; 7.2.

K_3[Al$_{0.99}$Cr$_{0.01}$(ox)$_3$] (5): Solid K_3[Cr(ox)$_3$]·3H_2O (0.09 mg, 0.0002 mmol) was added to a boiling aquous solution of K_3[Al(ox)$_3$]·4.5H_2O (9.68 mg, 0.0198 mmol) in 10 ml of H_2O, and stirring was maintained for 30 minutes. The solution was then kept standing and allowed to cool to room temperature. Upon slow evaporation, large block of crystals starts appearing in seven days. Metal analysis, ICP (% of M wrt Cr + Al, found): Al, 99.4; Cr, 0.6.

2. Physical Measurements

Elemental analyses were performed with an elemental microanalyzer (A5), model Flash 1112, at the Servei de Microanàlisi of CSIC, Barcelona, Spain. MALDI-TOF mass spectrometry measurements were performed in reflector positive mode, using a 4800 Plus MALDI TOF/TOF spectrometer ABSciex (Applied Biosystems) on the Biomolecular Analysis Unit (Mass Spectrometry for Molecular Characterization) from Scientific and Technological Centers of the University of Barcelona (CCiTUB). For these, each solid compound was solved in 0.2 mL MeOH:H_2O (1:1). Then 1µl of the sample solution was mixed with 1µl of the matrix solution DCTB (10mg/mL in dichloromethane) and 1µl of the resulting mixture was deposited on the plate and dried under nitrogen before analysis by MALDI-TOF. IR spectra were recorded in an Thermo Scientific™ Nicolet™ iS™5 FT-IR Spectrometer.

3. Single Crystal X-Ray Diffraction

Data for compounds **1** and **2** were collected at 100 K on the BL13-XALOC beamline[3] of the ALBA synchrotron (λ = 0.72932 Å) with the collaboration of ALBA staff. Data for compound **4** were acquired at 100 K at Beamline 12.2.1 of the Advanced Light Source (Berkeley, USA), on a Bruker D8 diffractometer equipped with a PHOTON II detector and using silicon (111) monochromated synchrotron radiation (λ = 0.7288 Å). Data reduction for **1** and **2** was done with autoproc package[4] and XDS.[5] Data reduction and absorption corrections for **4** were performed with respectively SAINT and SADABS.[6] All structures were solved by intrinsic phasing with SHELXT[7] and refined by full-matrix least-squares on F^2 with SHELXL.[8] In the three structures, a portion of the lattice solvent molecules were too diffuse/disordered to be modelled satisfactorily. The corresponding void spaces were thus analyzed and accounted for with PLATON/SQUEEZE,[9] the formulas reflecting the squeezed content.
All details can be found in CCDC 2416208-2416209-2416210 (**1**-**2**-**4**) which contain the supplementary crystallographic data for this paper. These data can be obtained free of charge from The Cambridge Crystallographic Data Center via https://summary.ccdc.cam.ac.uk/structure-summary-form. Crystallographic and refinement parameters are summarized in Table S1**,** while Tables S2 and S3 provide details of hydrogen bonds involving the guest anion and of the coordination environment of the metal ions, respectively.

[3] J. Juanhuix, F. Gil-Ortiz, G. Cuní, C. Colldelram, J. Nicolás, J. Lidón, E. Boter, C. Ruget, S. Ferrer and J. Benach, *J. Synchrotron Radiat.*, 2014, **21** , 679-689.
[4] C. Vonrhein, C. Flensburg, P. Keller, A. Sharff, O. Smart, W. Paciorek, T. Womack, and G. Bricogne, *Acta Cryst. D,* 2011, **67**, 293-302.
[5] W. Kabsch, *Acta Cryst. D* 2010, **66**, 125-132
[6] a) G. M. Sheldrick, *SAINT and SADABS*, 2012, Bruker AXS Inc., Madison, Wisconsin, USA; b) L. Krause, R. Herbst-Irmer, G. M. Sheldrick, D. Stalke, *J. Appl. Cryst.*, 2015, **48**, 3-10.
[7] G. M. Sheldrick, *Acta Cryst. A* 2015, **71**, 3-8.
[8] G. M. Sheldrick, *Acta Cryst. C* 2015, **71**, 3-8.
[9] A. L. Spek, *Acta Cryst. C* 2015, **71**, 9-18.

4. EPR spectroscopy

EPR measurements were carried out using a Bruker Elexsys E580 spectrometer operating in X-band, equipped with a Stinger cryogen-free variable temperature control system, a Flexline cryostat, and an ER 4118X-MD5 dielectric resonator. All samples were prepared in Suprasil Wilmad tubes with a 4 mm outer diameter.

Hahn echo decays were recorded using the sequence: *(π/2)–d_1–(π)–d_1–echo*, with d_1 incremented in 2 ns steps starting from 100 ns. Pulse lengths were 8 ns for π/2 and 16 ns for π. The total envelop of the spin echo was integrated.

Inversion recovery measurements employed a conventional three-pulse sequence: *(π)–d_1–(π/2)–d_2–(π)–d_2–echo*. The pulse lengths were 8 ns for π/2 and 16 ns for π; the inter-pulse delay d_2 was set to 400 ns, with an initial inversion delay d_1 of 400 ns.

We used the following *CPMG* variant: $(\pi/2)_{\phi1}$ – {τ–$(\pi)_{\phi2}$ – τ –echo – τ –$(\pi)_{\phi2}$ – τ –echo}$_N$, where each π pulse is followed by a fixed inter-pulse delay 2τ of 600 ns for echo generation. A total of 14 refocusing pulses were used. To suppress artifacts and ensure phase coherence, a following phase cycling scheme was applied. The excitation pulse $(\pi/2)_{\phi1}$ was cycled as [+x,+x,−x,−x], and the refocusing $(\pi)_{\phi2}$ pulses used a repeating pattern of [−y,+y,−y,+y]. Receiver phase was adjusted accordingly to match echo formation and suppress FID/background signals. The full transient signal from each shot was recorded using quadrature detection and subsequently processed via a custom Python script.

Rabi oscillations were recorded by applying a nutation microwave pulse of variable length, adding a constant delay of 10 ms much longer than T_m time and monitoring the echo intensity as a function of nutation pulse duration. The nutation pulse width was incremented in steps of 2 ns. Baseline correction (first-order polynomial), exponential windowing function, zero-padding, and Fourier transformation were applied to the time-domain signal. The resulting spectra were analyzed to extract Rabi frequencies. The dependence of the experimental Rabi frequency on the calculated nutation frequency (assuming 44 MHz at 0 dB attenuation) was evaluated to verify the expected microwave power scaling behavior.

Spectral simulations were performed using the EasySpin,[10] based on the spin Hamiltonian: $\hat{H} = \boldsymbol{g}\mu_\mathrm{B}\mathbf{B}\hat{\boldsymbol{S}} + D\hat{S}_z^2 + E(\hat{S}_x^2 - \hat{S}_y^2)$. Simulations included distributions in the axial zero-field splitting parameter *D* (D-strain) to account for inhomogeneities in the sample.

[10] Stoll, S.; Schweiger, A., EasySpin, a comprehensive software package for spectral simulation and analysis in EPR. *J. Magn. Reson.* **2006,** *178*, 42-55.

Estimate of average separation and dipolar couplings between paramagnetic ions in the solid lattice

To facilitate a qualitative comparison between the oxalate and helicate lattices, we employed a simplified cubic approximation for both crystal structures. While the actual unit cells are not cubic, this model provides a reasonable first-order estimate of average paramagnetic ion separation and associated dipolar couplings. The average separation between paramagnetic ions depends on their number density *n*, which is defined as the number of paramagnetic ions per unit volume. For a cubic lattice with a distance *d* between nearest neighbors, the volume occupied by each center is $v = d^3$. Therefore, the number density is $n = p / d^3$, here *p* is the population of paramagnetic centers (e.g., $p = 0.01$ for 1% dilution).

The mean distance, which represents the average separation between paramagnetic ions in the lattice, can be expressed as $d_{mean} = n^{-1/3}$. Substituting $n = p / d^3$, we obtain:

$d_{mean} = d * p^{(-1/3)}$

Neglecting the anisotropic part of the standard dipolar spin Hamiltonian (which, after integration, contributes only an additional constant) and using the estimate for the dipolar coupling constant $D = \frac{\mu_0}{4\pi}\frac{g_1\mu_B g_1\mu_B}{r^3}$, we calculate the following values for the expected degrees of dilution, using X-ray-derived distances between chromium centers of 0.71 nm and 1.23 nm for free oxalate and helicate compounds, respectively.

Sample	Dilution (p)	Distance in undiluted cristal (d, nm)	Mean distance (d_{mean} nm)	D, MHz	Dilution (p)
**5**	0.01	0.71	3.30	1.45	0.01
**4**	0.03	1.23	3.96	0.84	0.03
**3**	0.09	1.23	2.74	2.51	0.09

Thus, the crude estimates of both the average distance and the dipolar coupling constant indicate that the 9% helicate sample (**3**) is expected to have larger dipolar coupling constant values in comparison with 1% oxalate sample (**5**). This demonstrates that the mere difference in the distance between paramagnetic centers is insufficient to explain the longer T_m times observed for the helicate.

Note that while particularly close paramagnetic neighbors can contribute strong local dephasing, the observed phase memory time reflects a distribution of couplings across the entire ensemble. Therefore, the mean inter-spin distance offers a more representative metric for assessing average dipolar-induced decoherence in a randomly diluted lattice. This approach also enables consistent comparison across different host lattices and doping levels.

5. Magnetometry

Direct current (*dc*) measurements were performed with a commercial MPMS3 SQUID magnetometer hosted by the Physical Measurements Unit of the Servicio General de Apoyo a la Investigación-SAI, Universidad de Zaragoza. Variable temperature measurements were done in settle mode (*i.e.* the temperature is stabilized at each temperature) and using DC scan. The low temperature variable field measurements were done using VSM measurement mode with 2 s acquisition, also in settle mode (*i.e.* stabilizing the magnetic field at each field). The data were corrected for the contribution of the plastic sample holder, determined empirically. The sample diamagnetic contributions to the susceptibility were corrected using Pascal's constant tables.

Isothermal alternating current (*ac*) measurements were collected in the range $0.4 \leq \nu \leq$ 900 Hz with the MPMS3 SQUID magnetometer with a 4 Oe amplitude and completed with measurements in the range $10 \leq \nu \leq 10000$ Hz with the ACMS option of a commercial PPMS-Dynacool set-up with the same 4 Oe amplitude. In all magnetic measurements, the sample was in the form of a fine polycrystalline powder, with crystallite size in the 1-10 μm range as determined through SEM observations, *a priori* precluding the presence of significant size/shape phonon bottleneck.

The characteristic relaxation times τ (see Table S4) were extracted from the frequency dependence of the real and imaginary components of the *ac* susceptibility, χ' and χ'' respectively, using the following expressions corresponding to the generalized Debye model:

$$\chi'(\omega) = \chi_S + (\chi_T - \chi_S)\frac{1 + (\omega\tau)^{\beta}\cos\left(\frac{\pi\beta}{2}\right)}{1 + 2(\omega\tau)^{\beta}\cos\left(\frac{\pi\beta}{2}\right) + (\omega\tau)^{2\beta}}$$

$$\chi''(\omega) = (\chi_T - \chi_S)\frac{(\omega\tau)^{\beta}\sin\left(\frac{\pi\beta}{2}\right)}{1 + 2(\omega\tau)^{\beta}\cos\left(\frac{\pi\beta}{2}\right) + (\omega\tau)^{2\beta}}$$

in which ω is the angular frequency, χ_T the isothermal susceptibility, χ_S the adiabatic susceptibility and β describes the distribution of relaxation times.

6. Figures

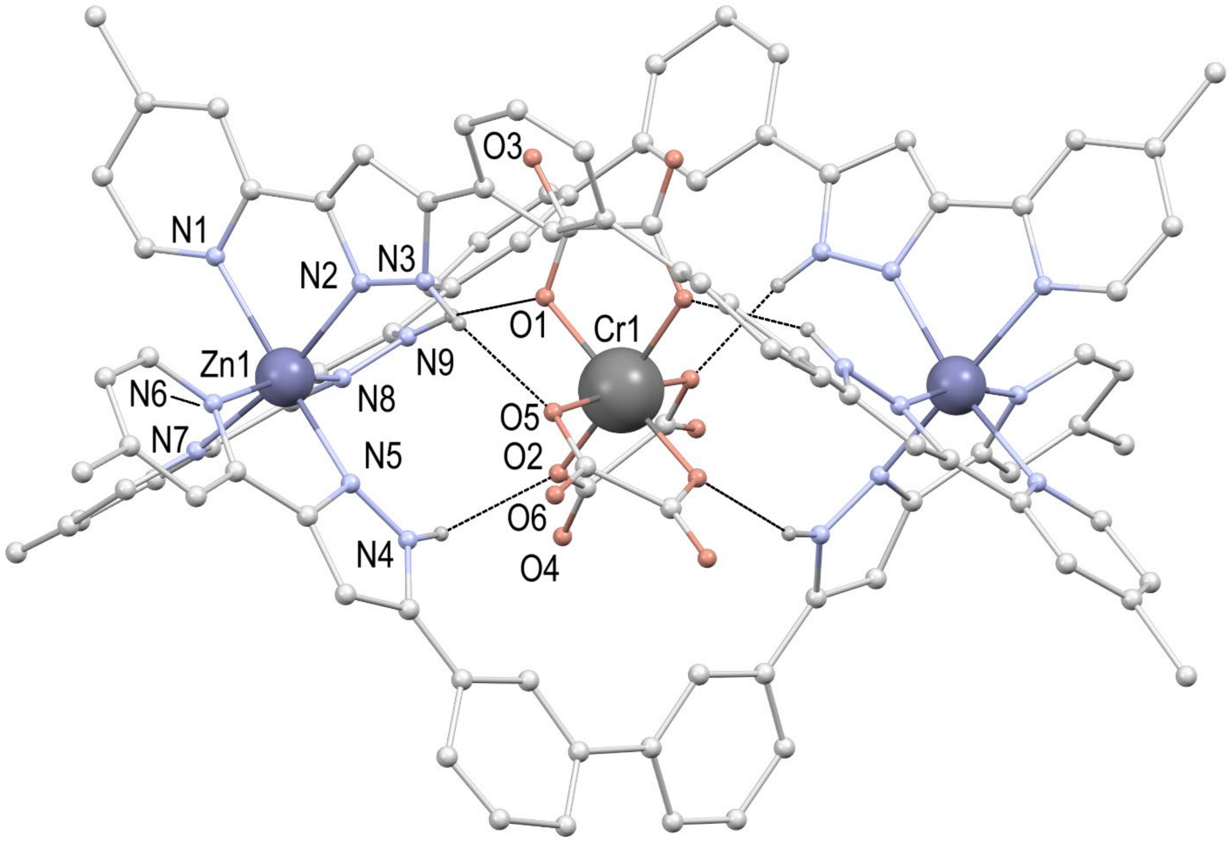

Figure S1. Molecular representation of $[Cr(ox)_3]@[Zn_2L_3]Cl$ (**1**) at 100K. Only heteroatoms are labelled. Only hydrogen atoms riding N atoms are shown (in small white spheres). Hydrogen bonds are emphasized with dashed lines.

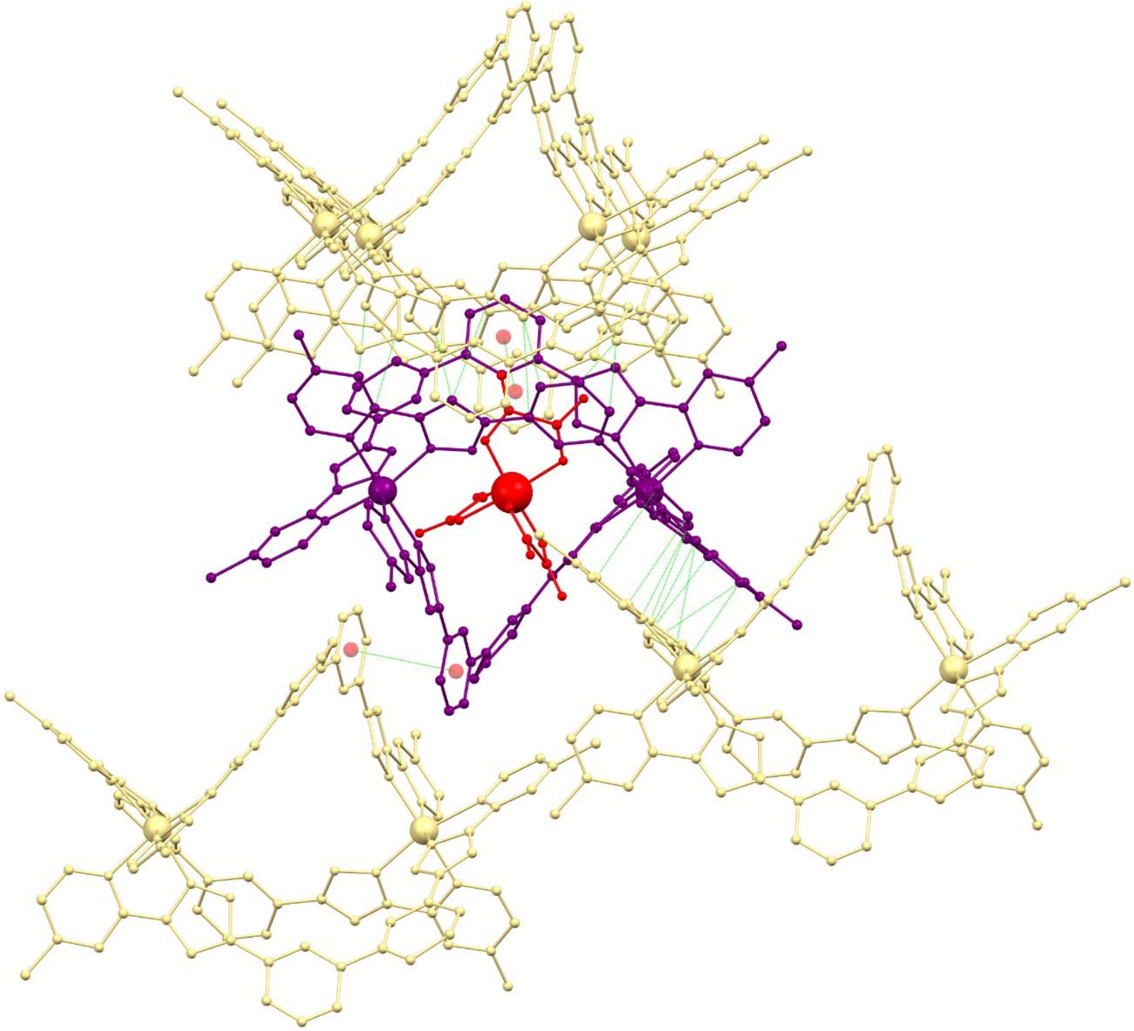

Figure S2. Representation of the crystal lattice of **1** with one $[Cr(ox)_3]@[Zn_2L_3]^+$ cationic assembly ($[Cr(ox)_3]^{3+}$ guest in red and $[Zn_2L_3]^{4+}$ host in purple) together with its four closest $[Zn_2L_3]^{4+}$ neighbors (in yellow); showing with dashed green lines the intermolecular interactions with these neighbors. The two types of interactions are either single-ring $\pi \cdots \pi$ contacts (centroids shown as red balls) or multiple $p\pi \cdots p\pi$ contacts.

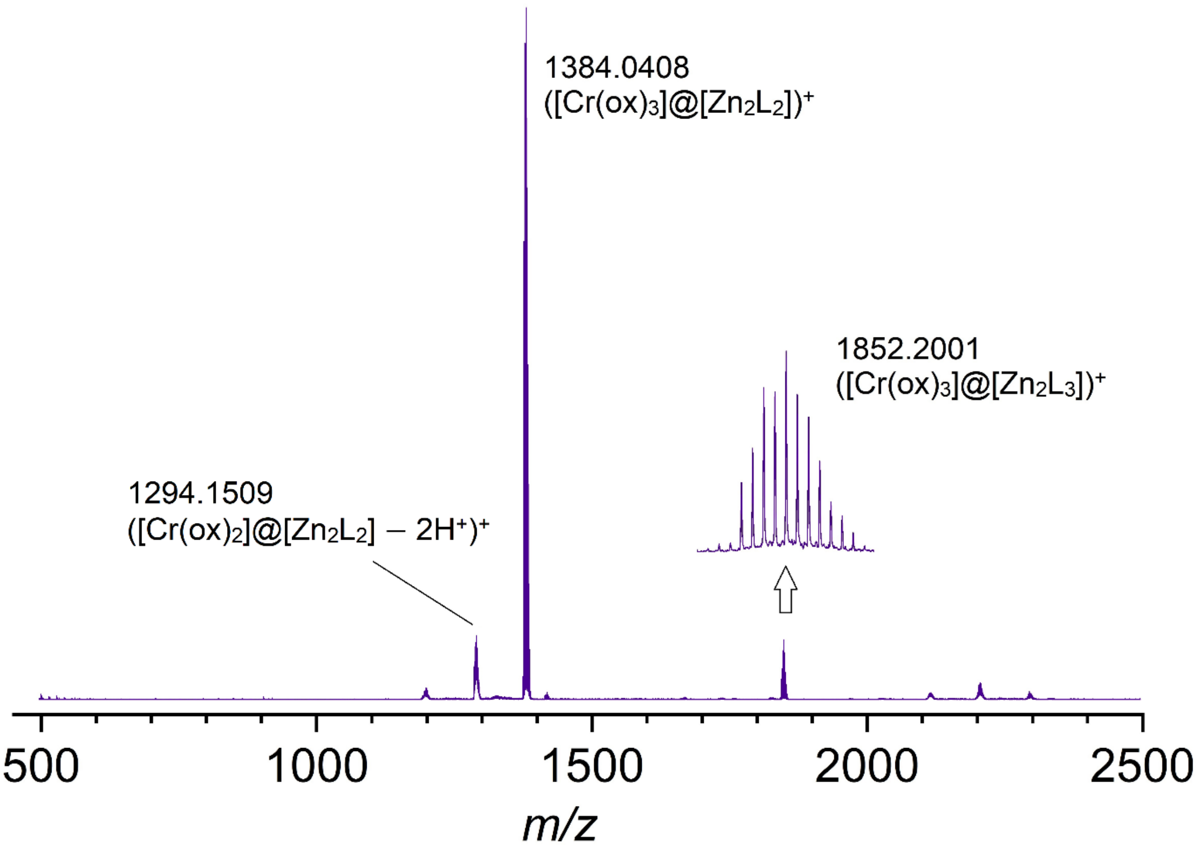

Figure S3. MALDI-TOF spectrum of compound **1** ($[Cr(ox)_3]@[Zn_2L_3]Cl$) in a matrix solution of DCTB with H_2O and MeOH (1:1), emphasizing the most important peaks, including the main cationic unit $(Cr(ox)_3]@[Zn_2L_3])^+$.

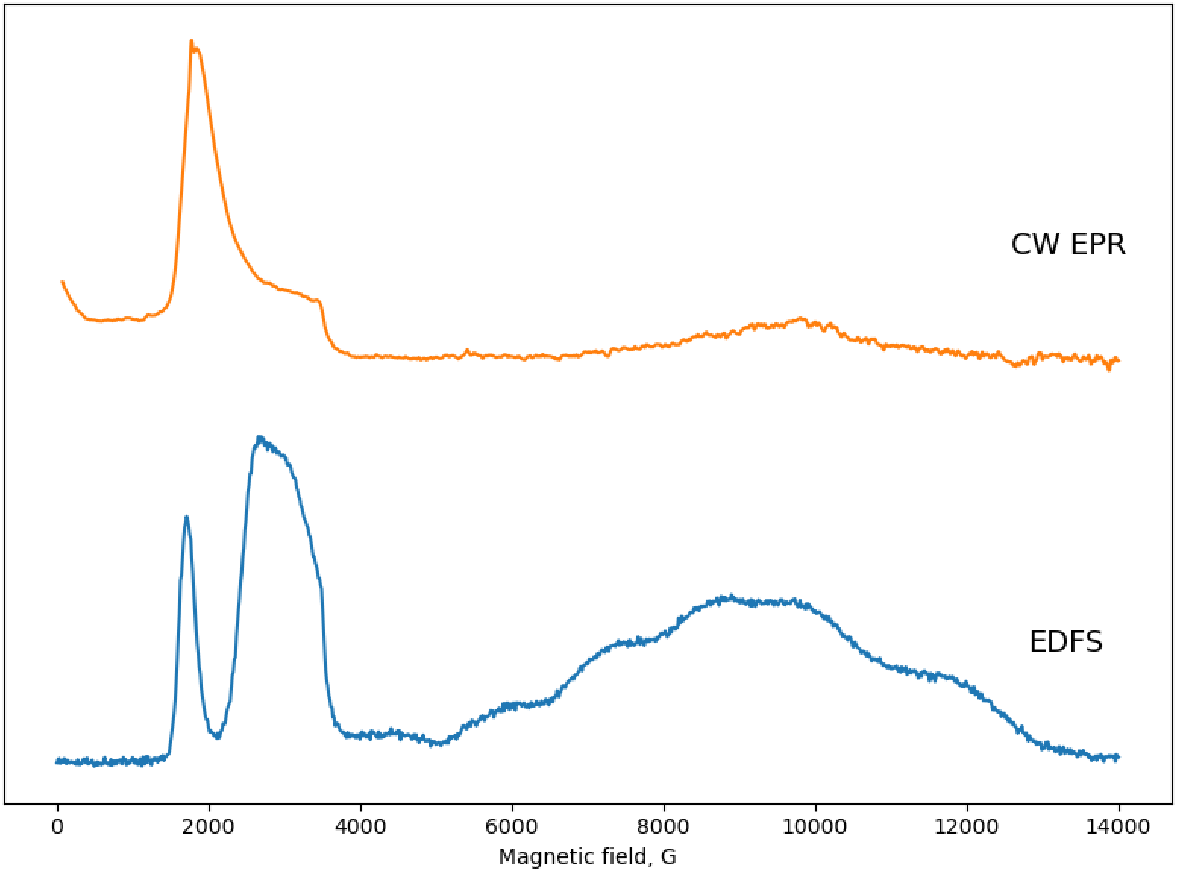

Fig. S4. EPR spectra of a 1 mM DMF solution of **1** at 4 K. (Top) CW EPR spectrum recorded with a microwave power of 10 dB and a modulation amplitude of 10 G. The spectrum is saturated, as it was not possible to obtain a good signal-to-noise ratio within a reasonable accumulation time at lower microwave power. (Bottom) Echo-detected field-sweep EPR spectrum.

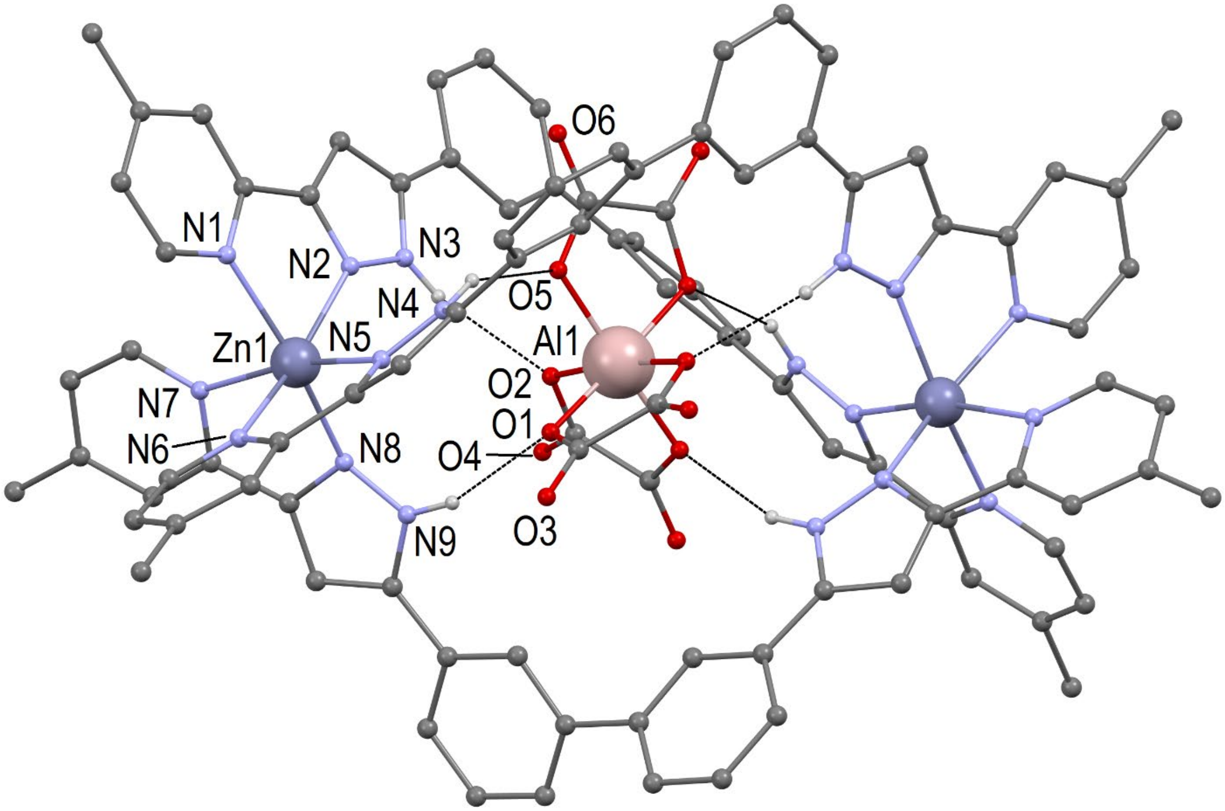

Figure S5. Molecular representation of [Al(ox)$_3$]@[Zn$_2$L$_3$]Cl (**2**) at 100K. Only heteroatoms are labelled. Only hydrogen atoms riding N atoms are shown (in small white spheres). Hydrogen bonds are emphasized with dashed lines.

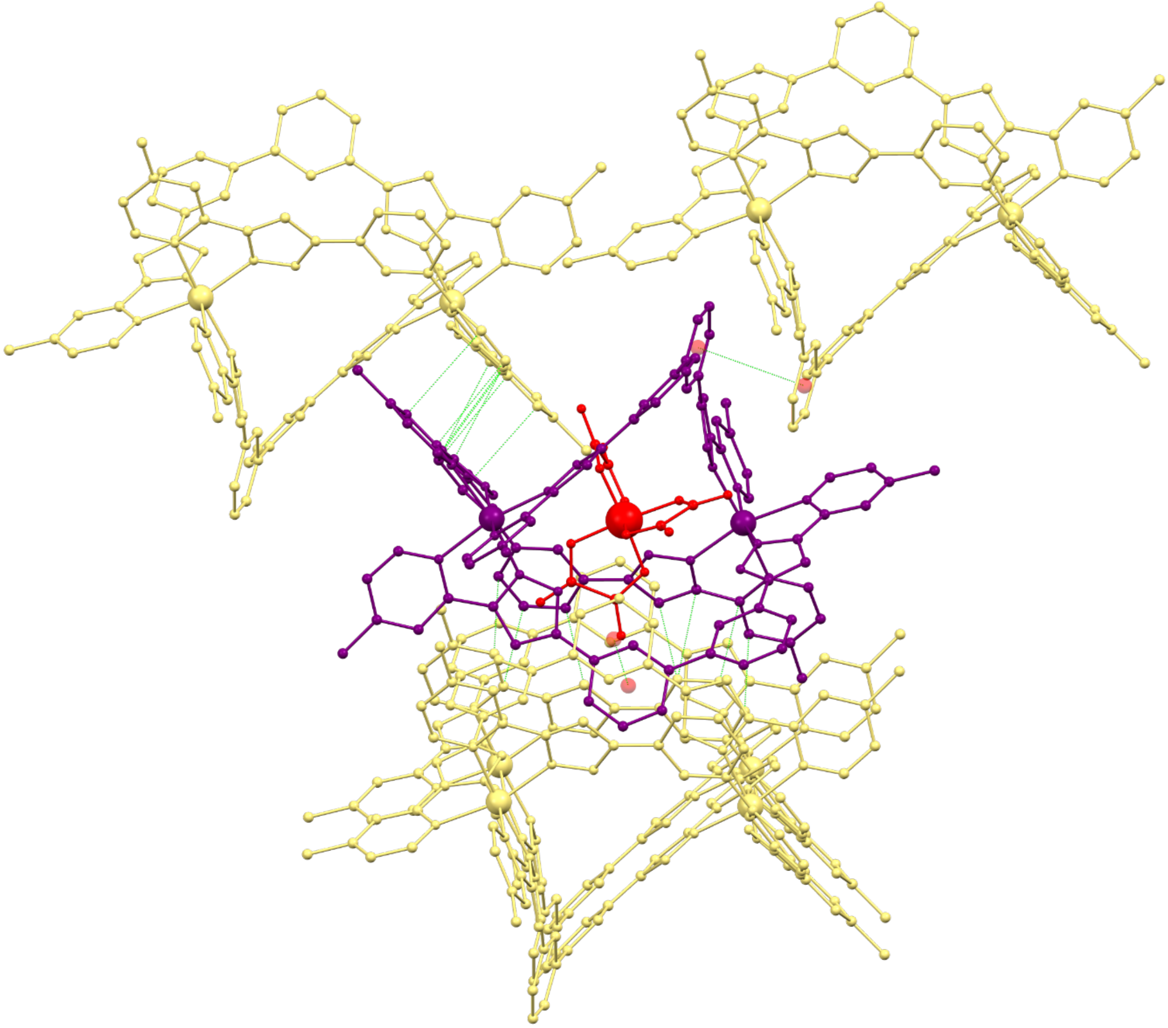

Figure S6. Representation of the crystal lattice of **2** with one $[Al(ox)_3]@[Zn_2L_3]^+$ cationic assembly ($[Al(ox)_3]^{3+}$ guest in red and $[Zn_2L_3]^{4+}$ host in purple) together with its four closest $[Zn_2L_3]^{4+}$ neighbors (in yellow) showing with dashed green lines the intermolecular interactions with these neighbors. The two types of interactions are either single-ring $\pi\cdots\pi$ contacts (centroids shown as red balls) or multiple $p\pi\cdots p\pi$ contacts.

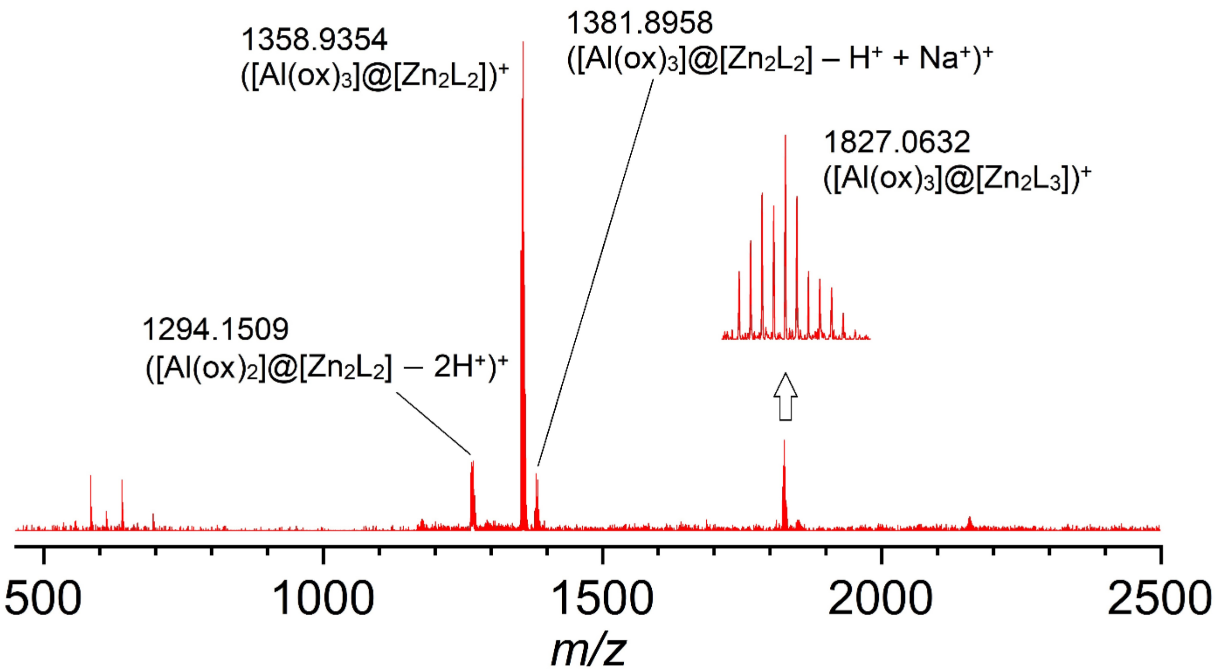

Figure S7. MALDI-TOF spectrum of compound **2** ($Al(ox)_3]@[Zn_2L_3]Cl$) in a matrix solution of DCTB with H_2O and MeOH (1:1), emphasizing the most important peaks, including the main cationic unit ($Al(ox)_3]@[Zn_2L_3]$)$^+$.

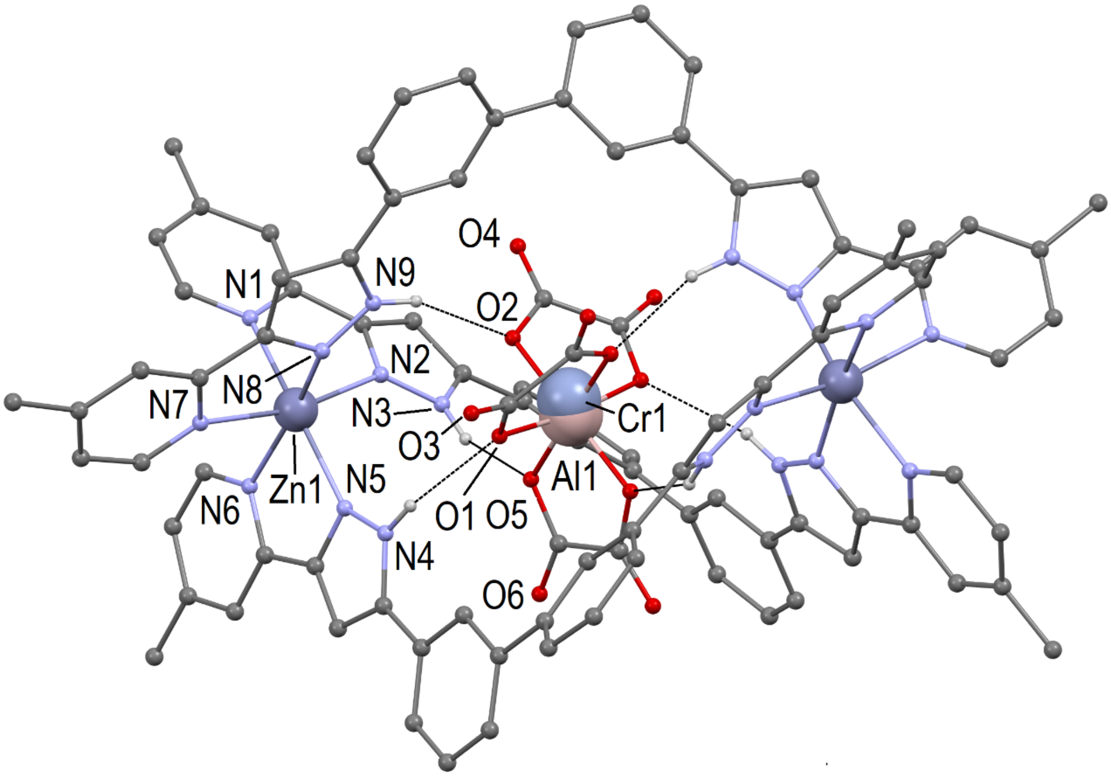

Figure S8. Molecular representation of ($[Al(ox)_3]_{0.97}[Cr(ox)_3]_{0.03}$)@$[Zn_2L_3]Cl$ (**4**) at 100K. The positions of Cr and Al do not coincide (they are 0.292 Å apart) and both are shown simultaneously. Only heteroatoms are labelled. Only hydrogen atoms riding N atoms are shown (in small white spheres). Hydrogen bonds are emphasized with dashed lines.

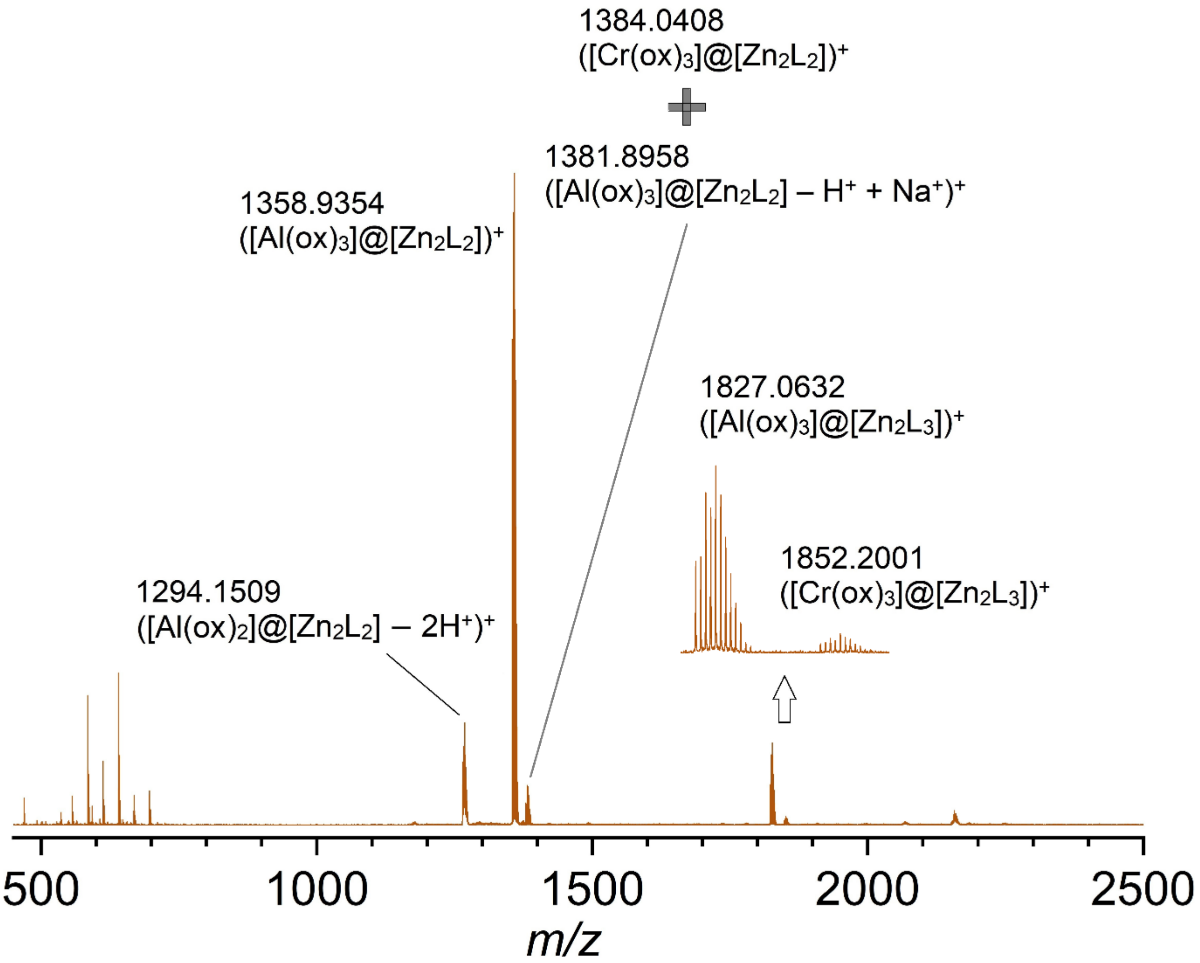

Figure S9. MALDI-TOF spectrum of compound **3** ($[Al(ox)_3]_{0.91}[Cr(ox)_3]_{0.09})@[Zn_2L_3]Cl$) in a matrix solution of DCTB with H_2O and MeOH (1:1), emphasizing the most important peaks, including the main cationic units ($Cr(ox)_3]@[Zn_2L_3])^+$ and ($Al(ox)_3]@[Zn_2L_3])^+$.

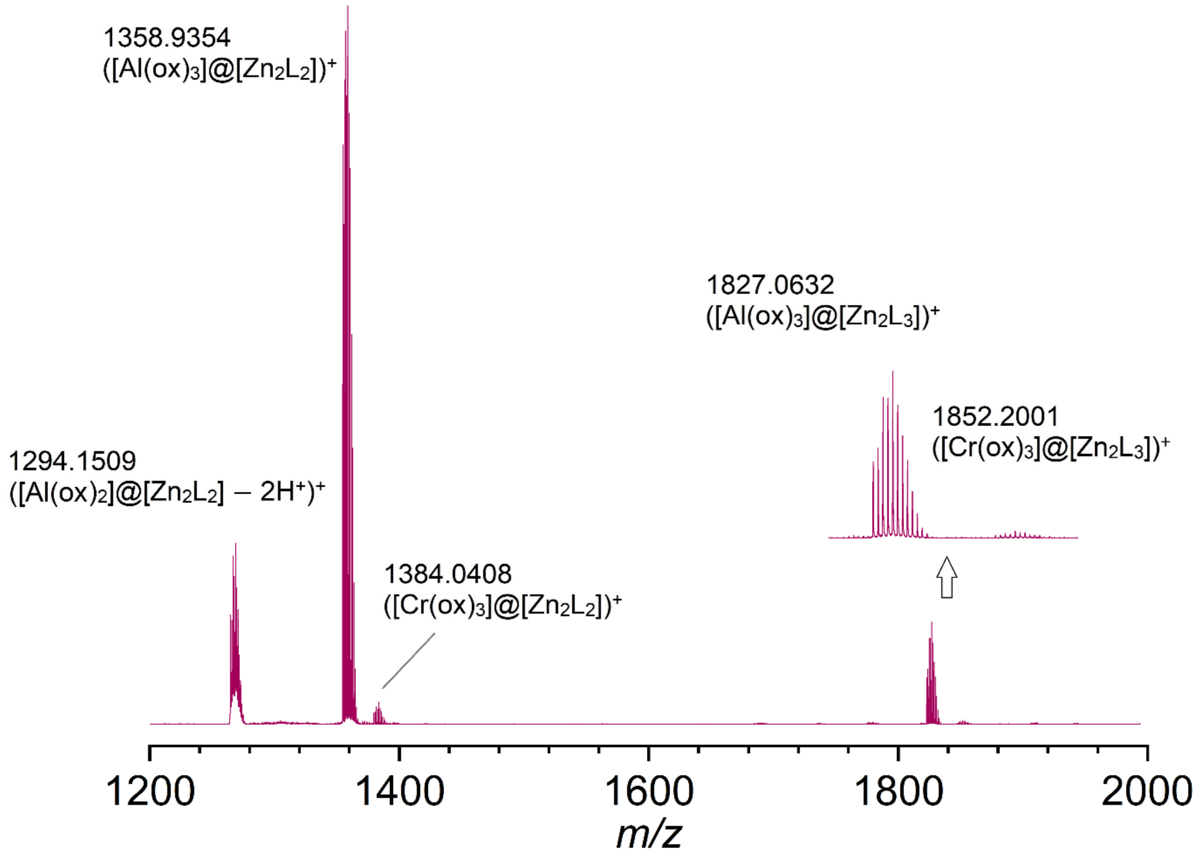

Figure S10. MALDI-TOF spectrum of compound **4** ($[Al(ox)_3]_{0.97}[Cr(ox)_3]_{0.03})@[Zn_2L_3]Cl$) in a matrix solution of DCTB with H_2O and MeOH (1:1), emphasizing the most important peaks, including the main cationic units ($Cr(ox)_3]@[Zn_2L_3])^+$ and ($Al(ox)_3]@[Zn_2L_3])^+$.

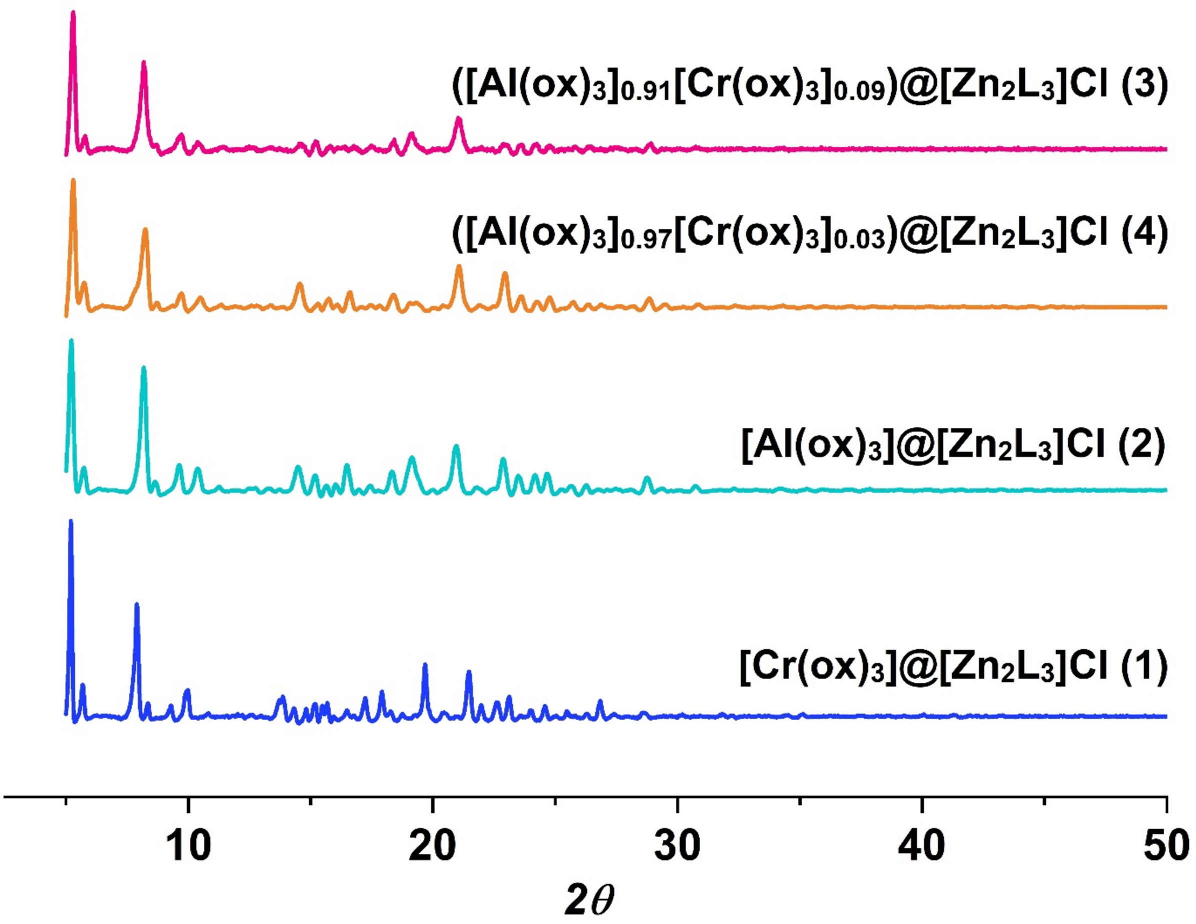

Figure S11. PXRD diffractograms of compounds **1** to **4** near 300 K showing their isostructural nature.

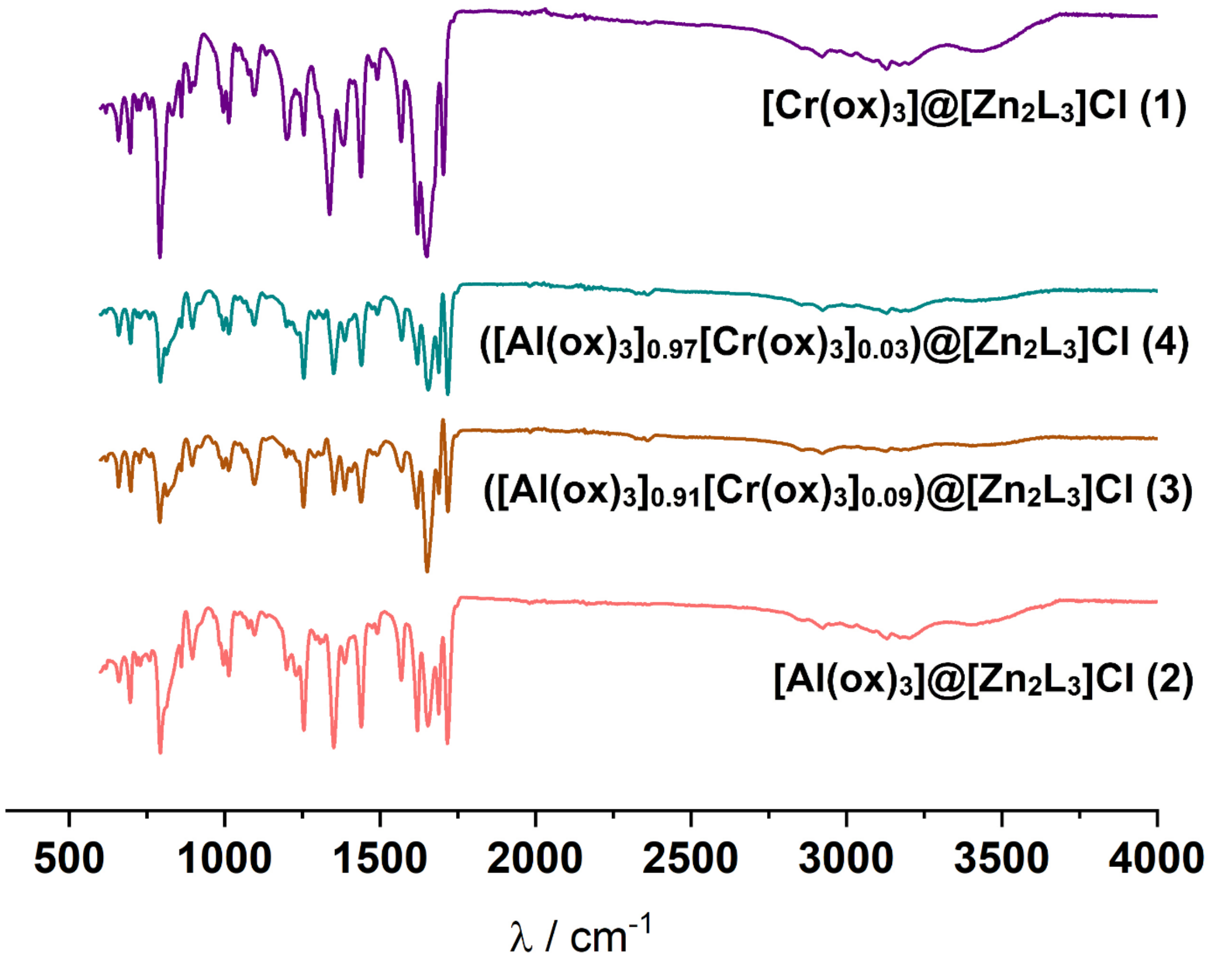

Figure S12. IR plots for compounds **1** to **4** near 300 K showing their isostructural nature.

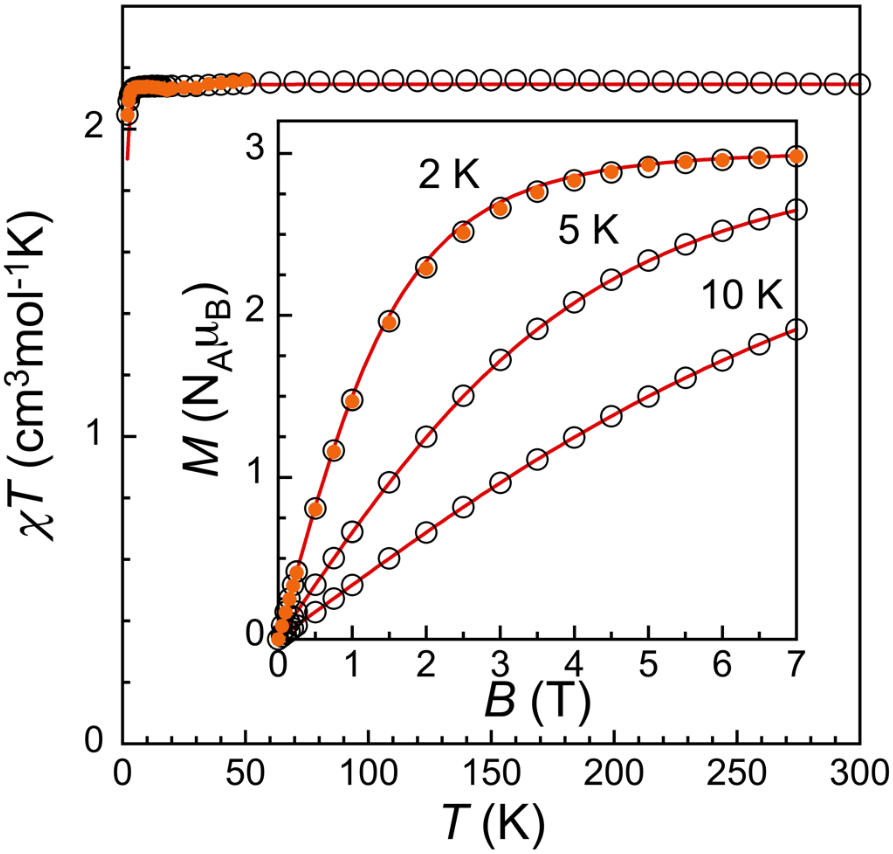

Figure S13. Temperature dependence of χT for compounds **1** (empty large black symbols) and **3** (full smaller red symbols) as derived from *dc* measurements at 0.5 T. Inset: magnetization isotherms at 2, 5 and 10 K for compound **1** and **3** (full smaller red symbols, only 2K data). Full red lines are the corresponding magnetization data calculated with Easyspin using the parameters reproducing the CW and EDFS EPR spectra. The data for **3** are shown after scaling for a composition Al:Cr of 0.94:0.06.

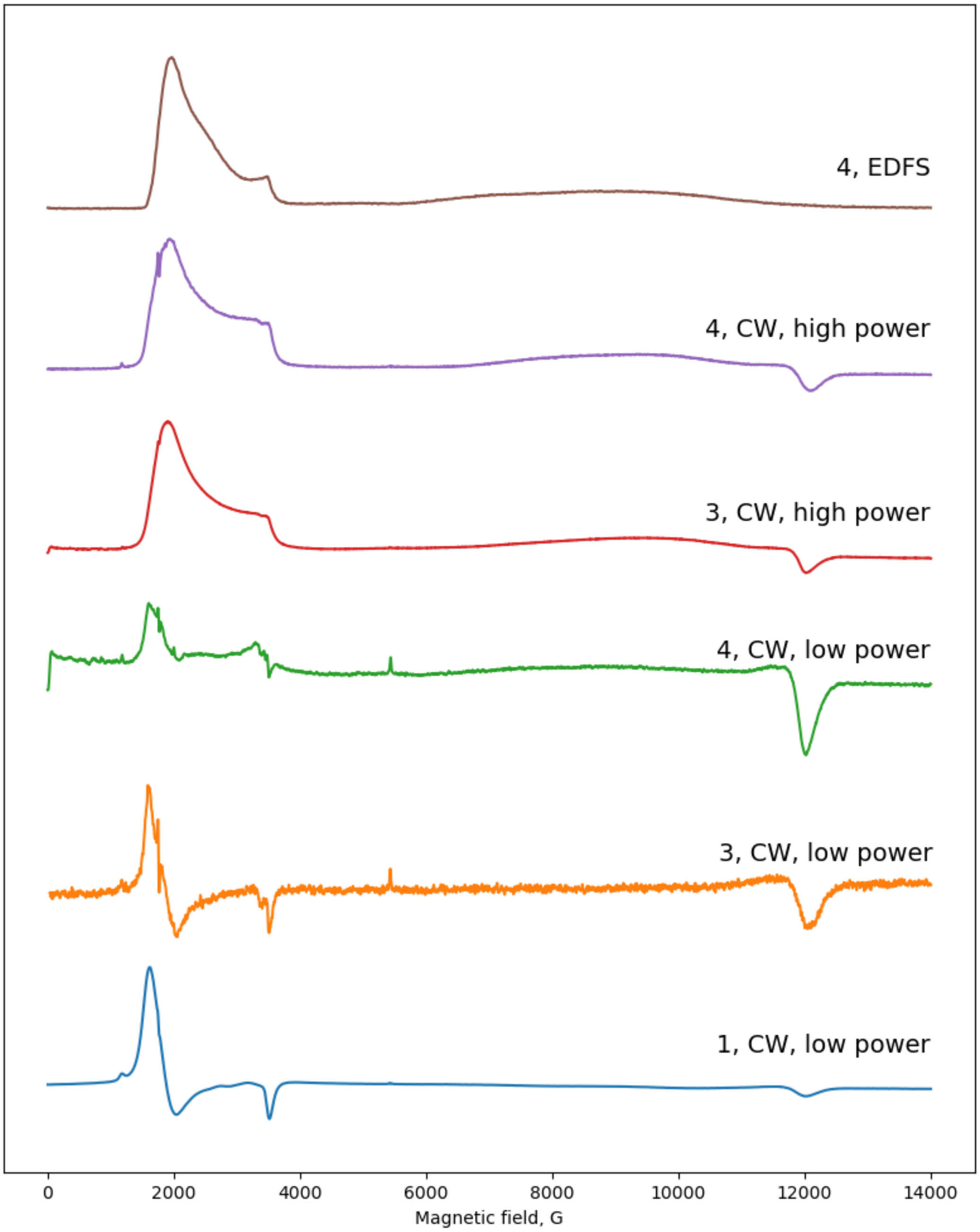

Figure S14. Comparison of solid-state EPR spectra of **1**, **3**, and **4** at 4 K under different measurement conditions. Low-power CW spectra were recorded with a microwave power attenuation of 50 dB, and high-power spectra at 30 dB. The narrow signals, as well as the broad signal around 12,000 G, originate from the resonator cavity. Note that the saturated high-power spectra are nearly identical to the echo-detected field-sweep (EDFS) spectrum.

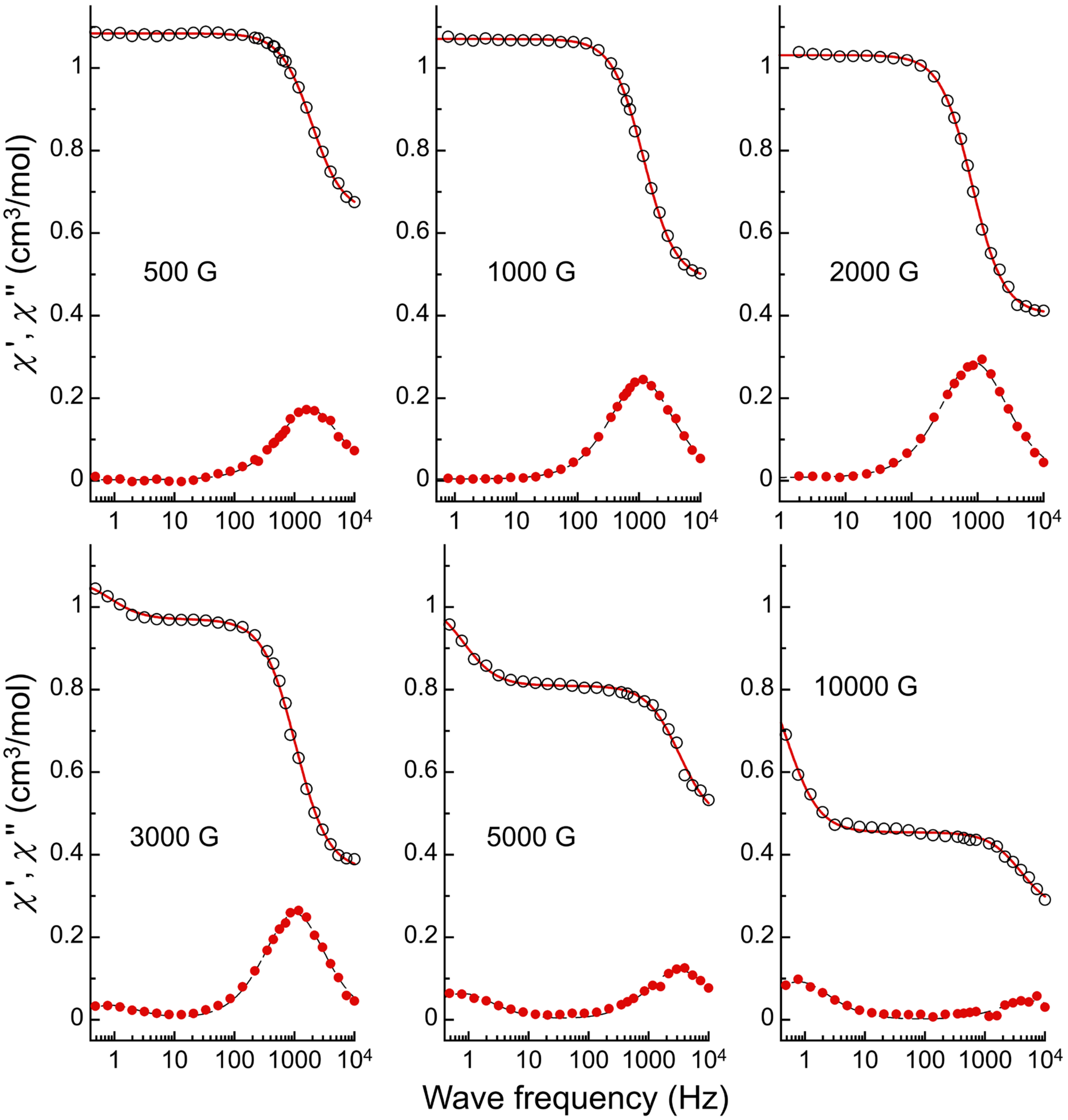

Figure S15. Frequency dependence of the in-phase (empty black symbols) and out-of-phase (full red symbols) *ac* magnetic susceptibility of compound **1** at 2 K and increasing applied *dc* fields as indicated. Lines are fits of the experimental data to the Cole-Cole expressions for the real and imaginary susceptibility.

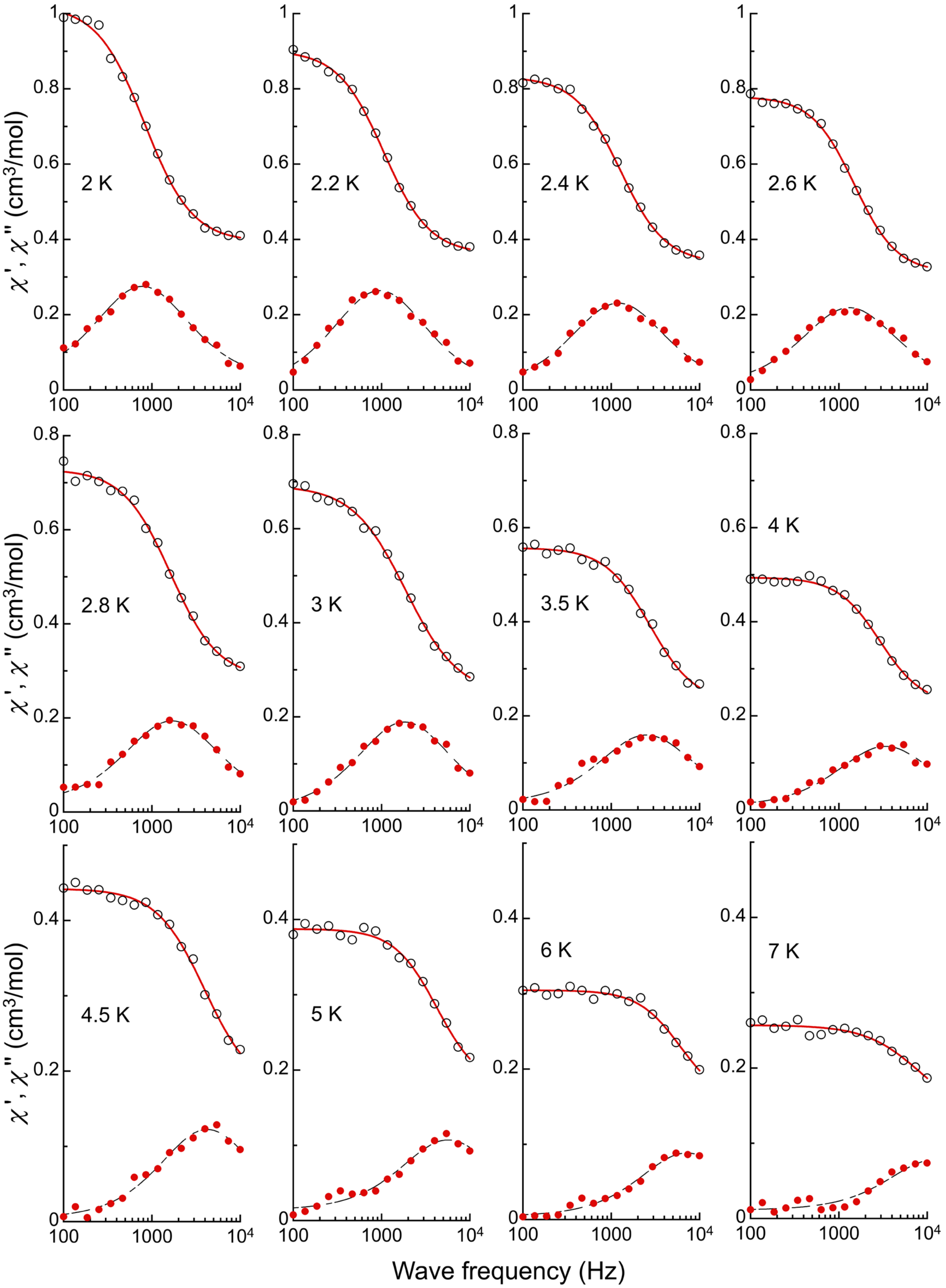

Figure S16. Frequency dependence of the in-phase (empty black symbols) and out-of-phase (full red symbols) *ac* magnetic susceptibility of compound **1** at 2000 G applied *dc* field and indicated temperatures. Lines are fits of the experimental data to the Cole-Cole expressions for the real and imaginary susceptibility.

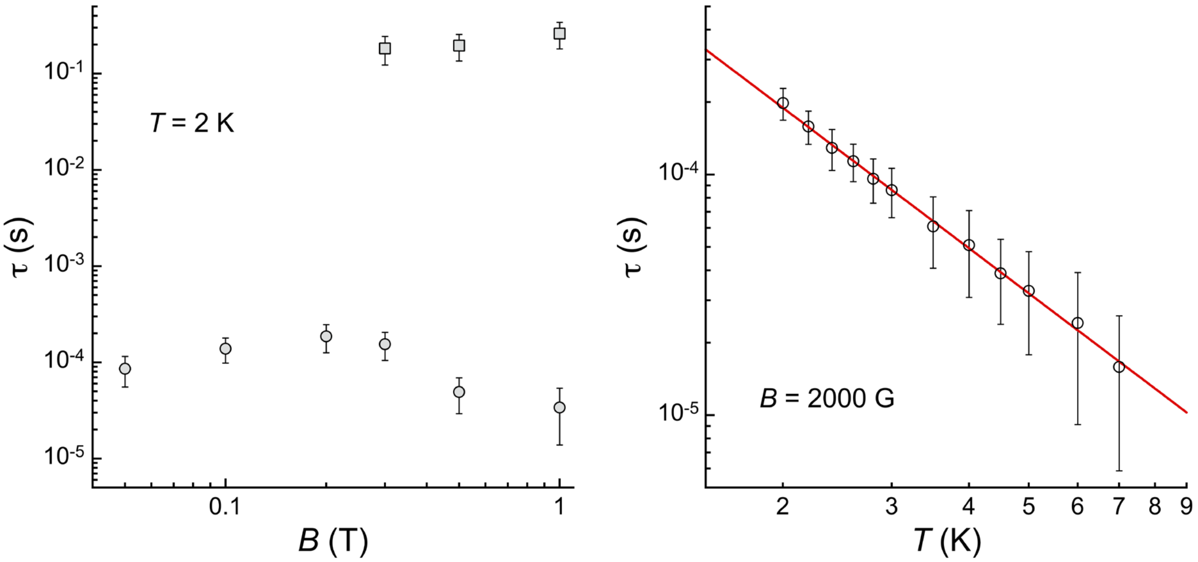

Figure S17. Left: Field dependence at T = 2 K of the characteristic relaxation times τ derived from the frequency dependence of the ac susceptibility for compound **1**. Right: Temperature dependence of the characteristic relaxation times τ of **1** under an applied *dc* field of 2000 Oe. The full red line is a fit to a power law, $\tau = 0.000725{*}T^{-1.94}$ yielding R = 0.9986.

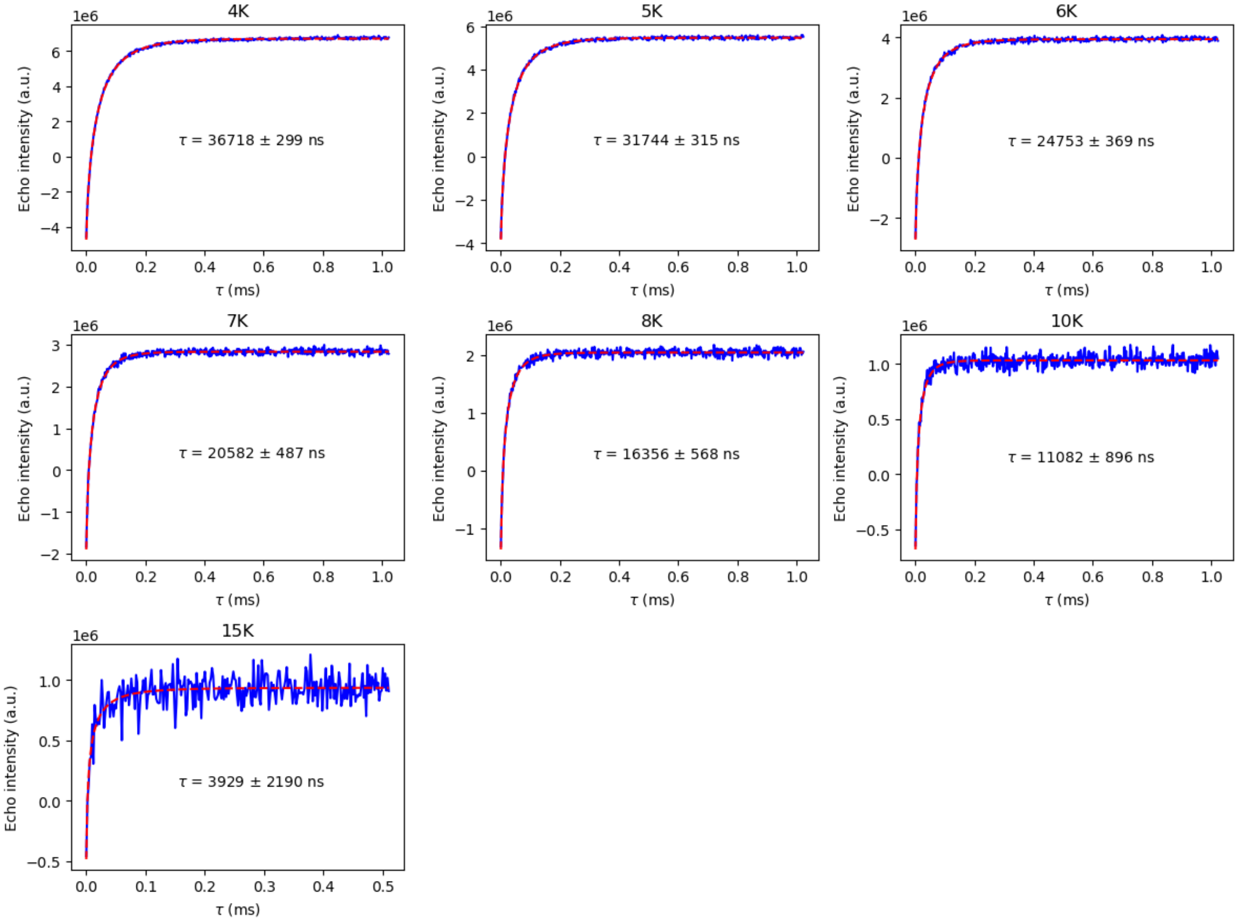

Figure S18. Inversion recovery experiments for **3** at various temperatures (4 – 15 K) in a magnetic field of 3480 G. Spin-lattice relaxation times of $[Cr(ox)_3]^{3-}$ were estimated by fitting the inverted data with a stretched exponential decay function.

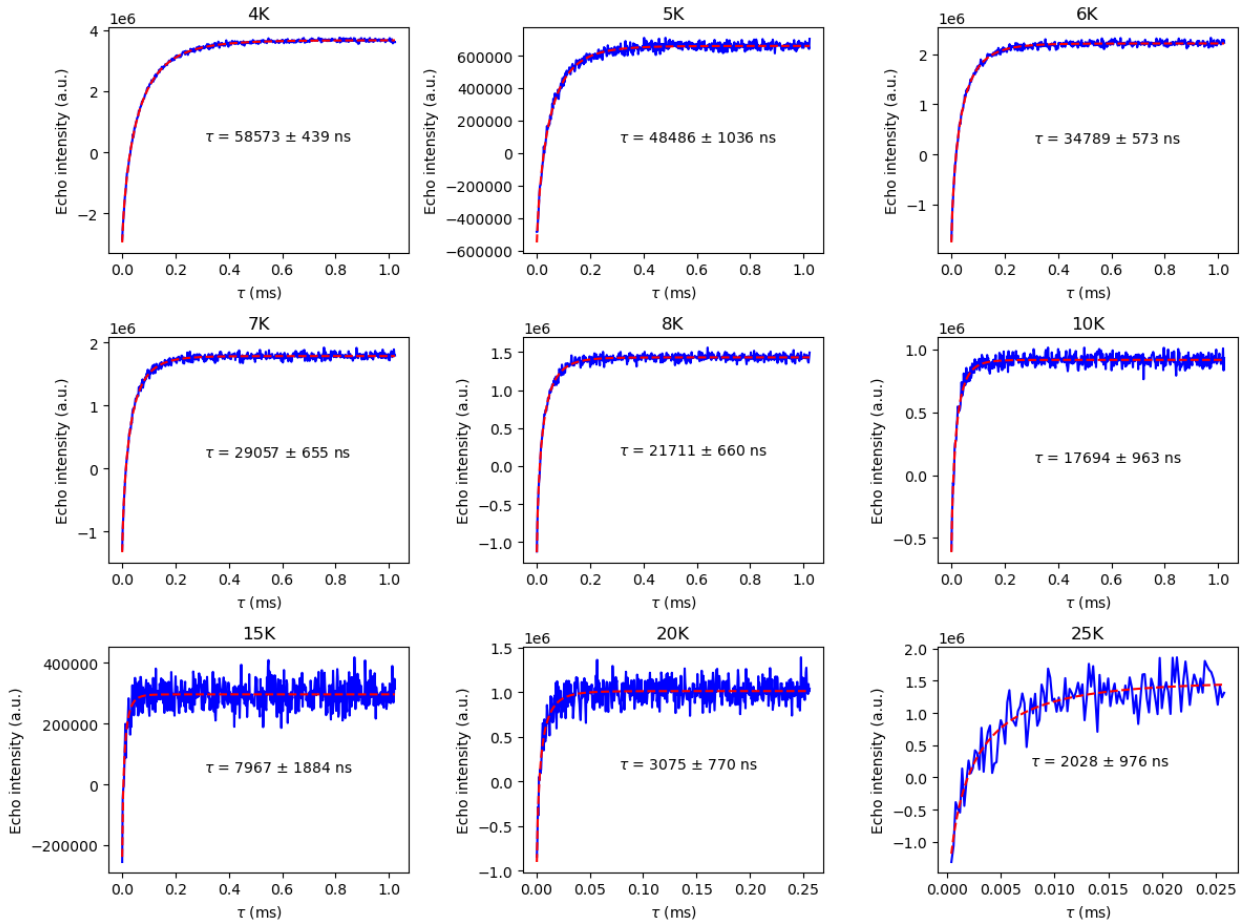

Figure S19. Inversion recovery experiments for **4** at various temperatures (4 – 25 K) in a magnetic field of 3480 G. Spin-lattice relaxation times of $[Cr(ox)_3]^{3-}$ were estimated by fitting the inverted data with a stretched exponential decay function.

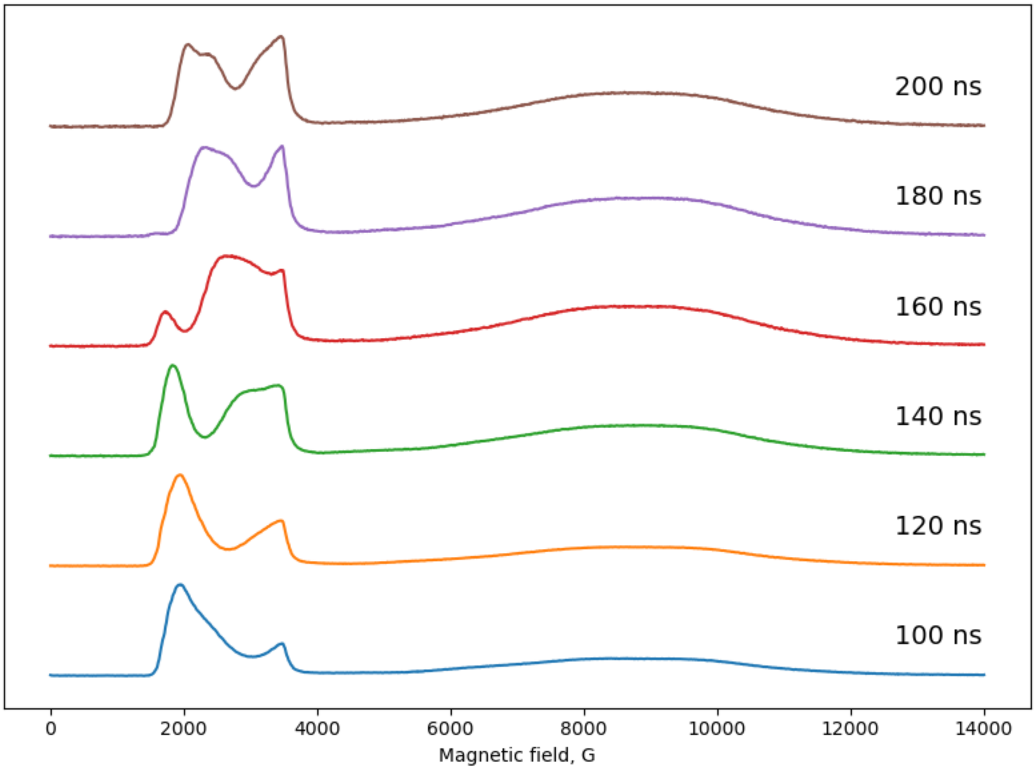

Fig. S20. EDFS spectra of **3** at 5 K recorded with different values of the interpulse delay τ. The strong dependence of the spectral lineshape on τ is attributed to a pronounced ESEEM effect.

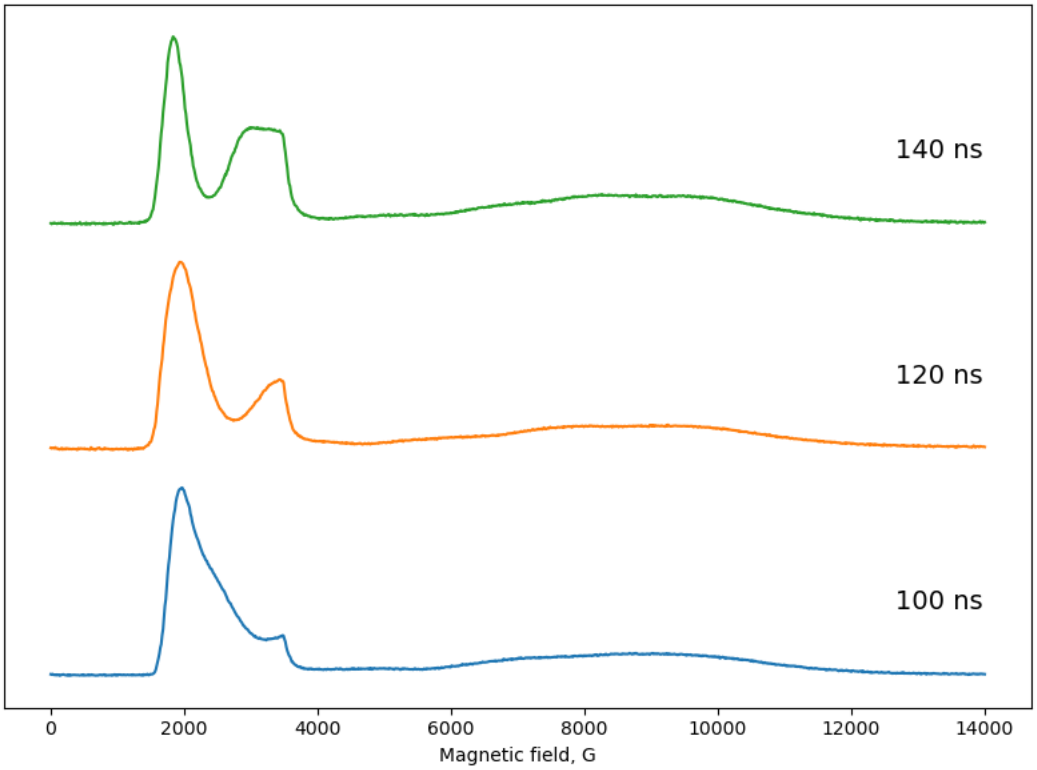

Fig. S21. EDFS spectra of **4** at 5 K recorded with different values of the interpulse delay τ. The strong dependence of the spectral lineshape on τ is attributed to a pronounced ESEEM effect.

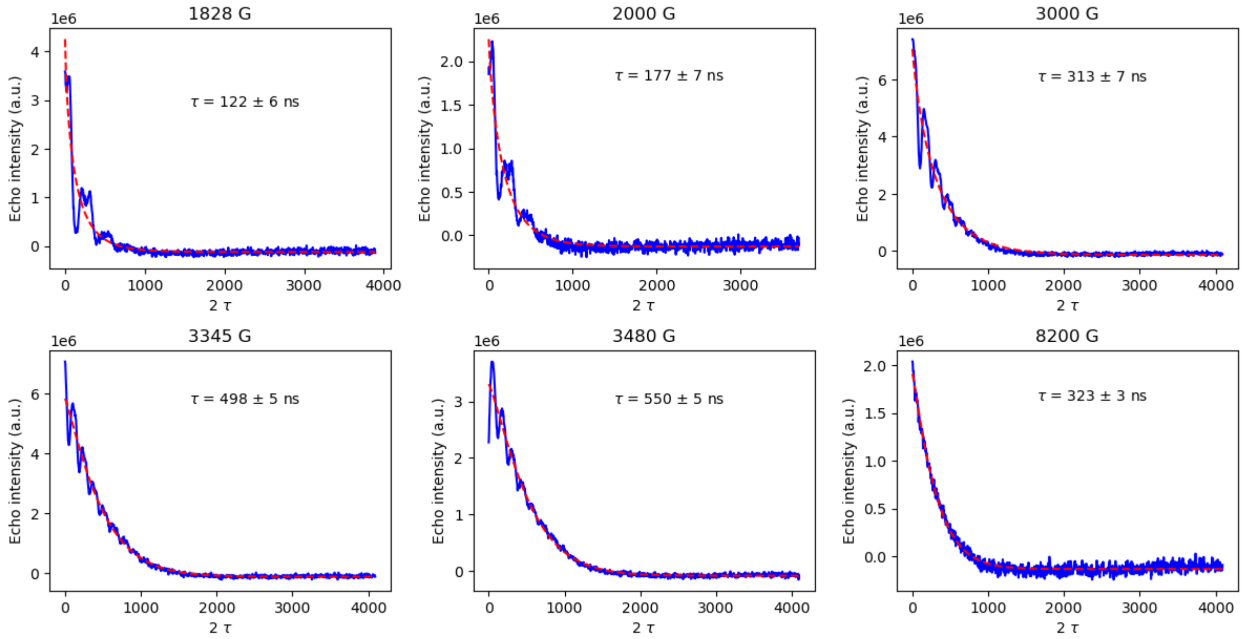

Figure S22. Hahn-echo decay of **4** at 10 K at different magnetic fields. Phase memory times were estimated by fitting the data with a stretched exponential decay function.

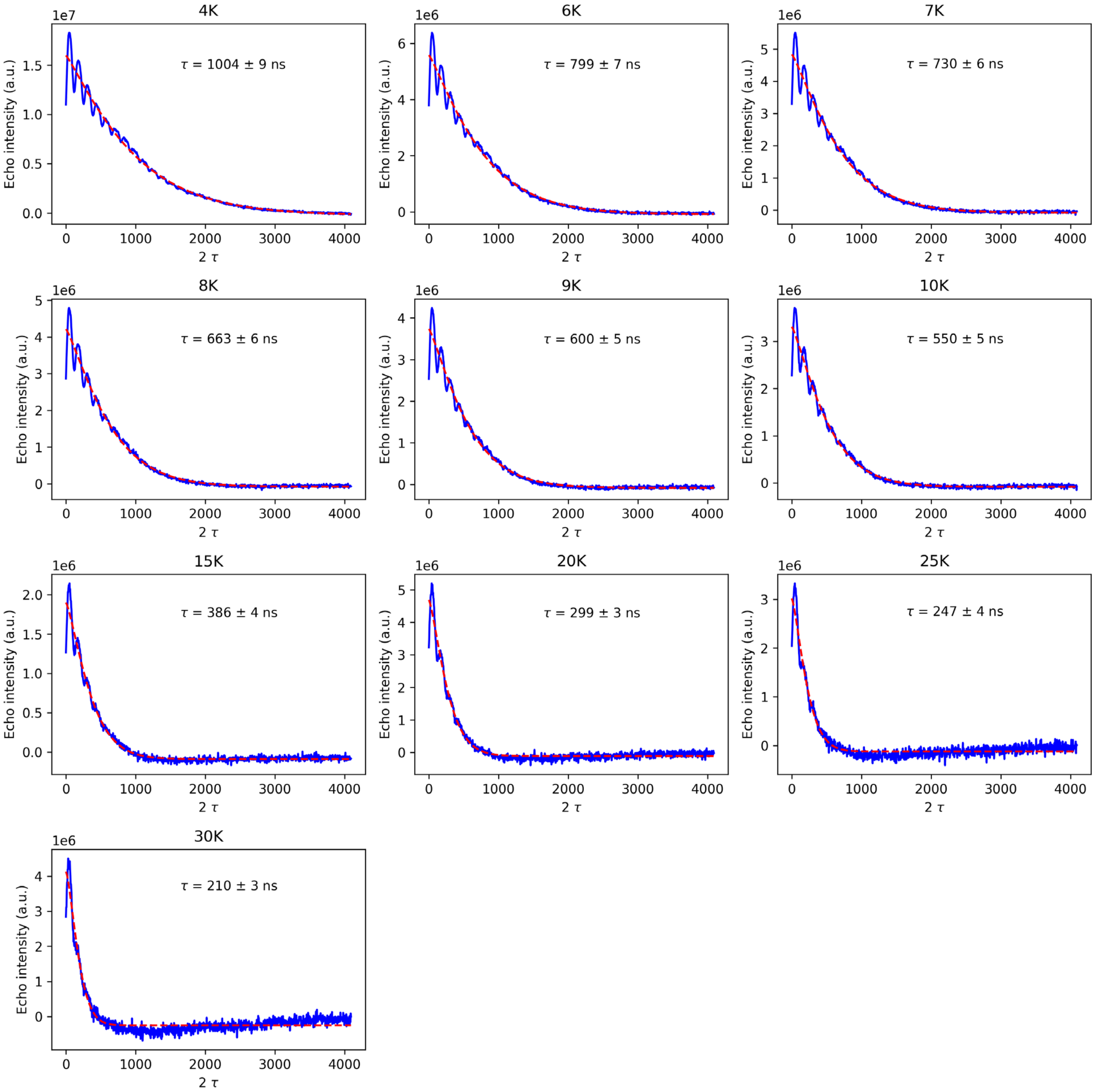

Figure S23. Hahn-echo decay of **4** at a magnetic field of 3480 G and various temperatures. Phase memory times were estimated by fitting the data with a stretched exponential decay function.

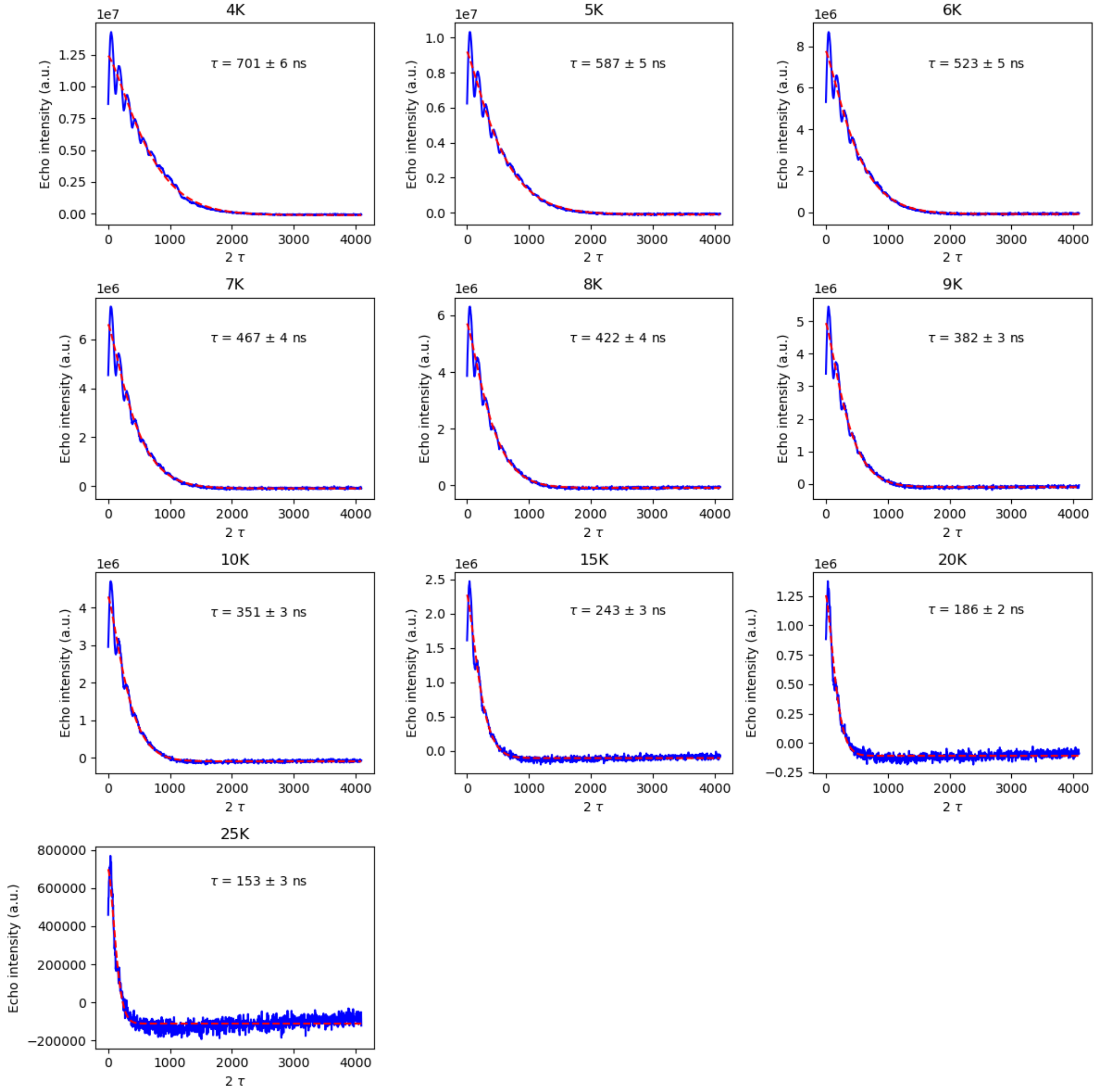

Figure S24. Hahn-echo decay of **3** at a magnetic field of 3480 G and various temperatures. Phase memory times were estimated by fitting the data with a stretched exponential decay function.

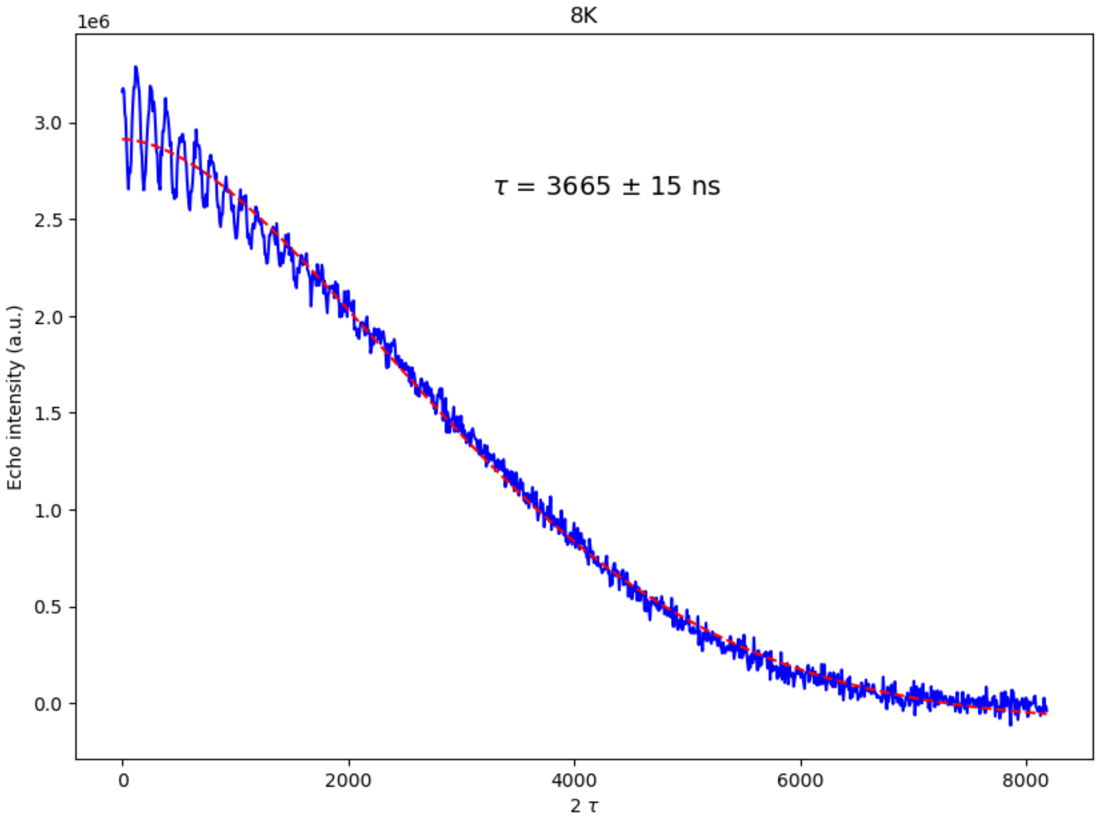

Figure S25. Hahn-echo decay of 1 mM solution of $K_3[Cr(ox)_3]$ in H_2O:glycerol (1:1) at a magnetic field of 3480 G and 8K. Phase memory time was estimated by fitting the data with a stretched exponential decay function.

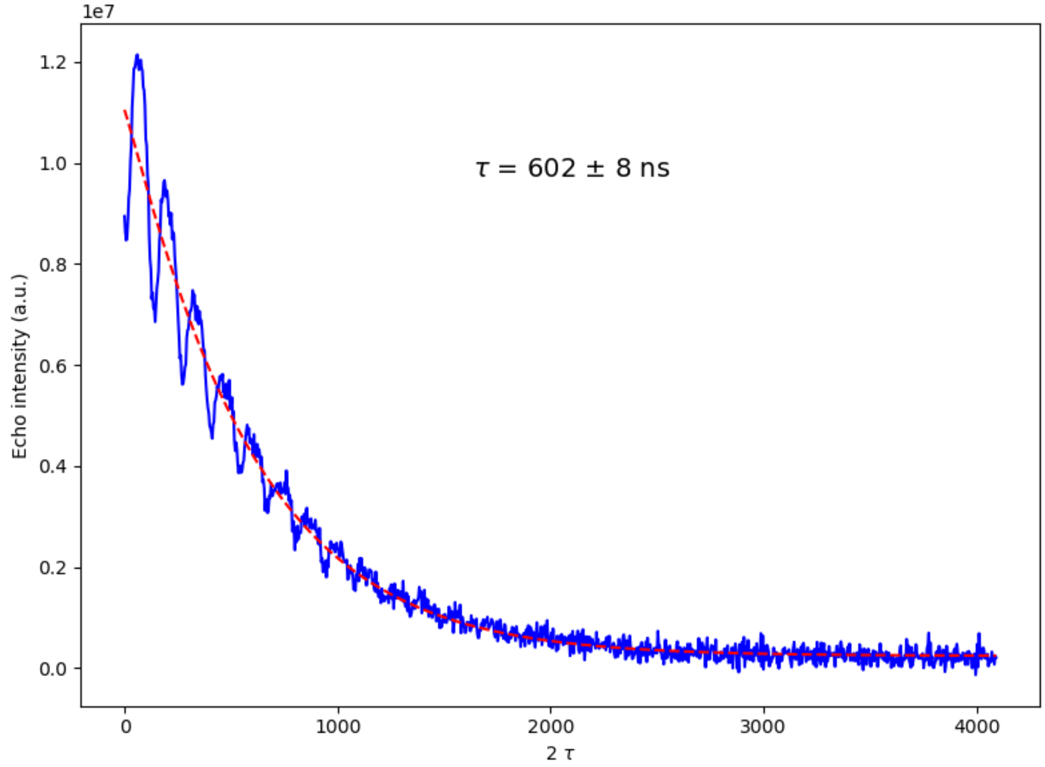

Fig. S26. Hahn-echo decay of a 1 mM DMF solution of **1** at 4 K and a magnetic field of 3480 G. The phase memory time was estimated by fitting the data with a stretched exponential decay function.

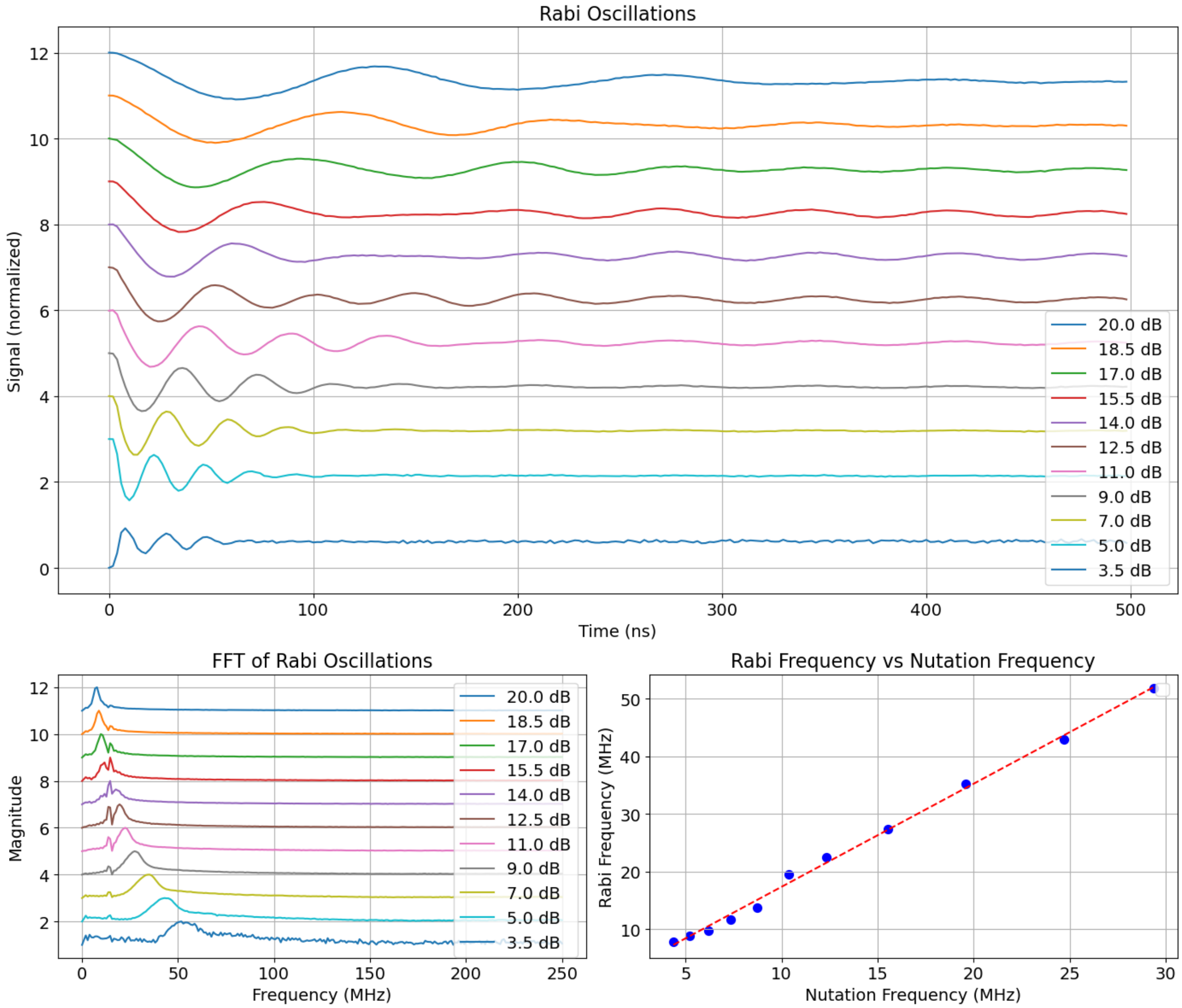

Figure S27. *(Top)* Rabi oscillations for compound **4** measured at 4 K and a magnetic field of 3480 G under different microwave power attenuation levels. *(Bottom left)* Corresponding Fourier transforms, showing an additional narrow peak attributed to the ^{1}H ESEEM effect. *(Bottom right)* Dependence of the experimental Rabi frequency on the nutation frequency, calculated assuming a nutation frequency of 44 MHz at 0 dB attenuation.

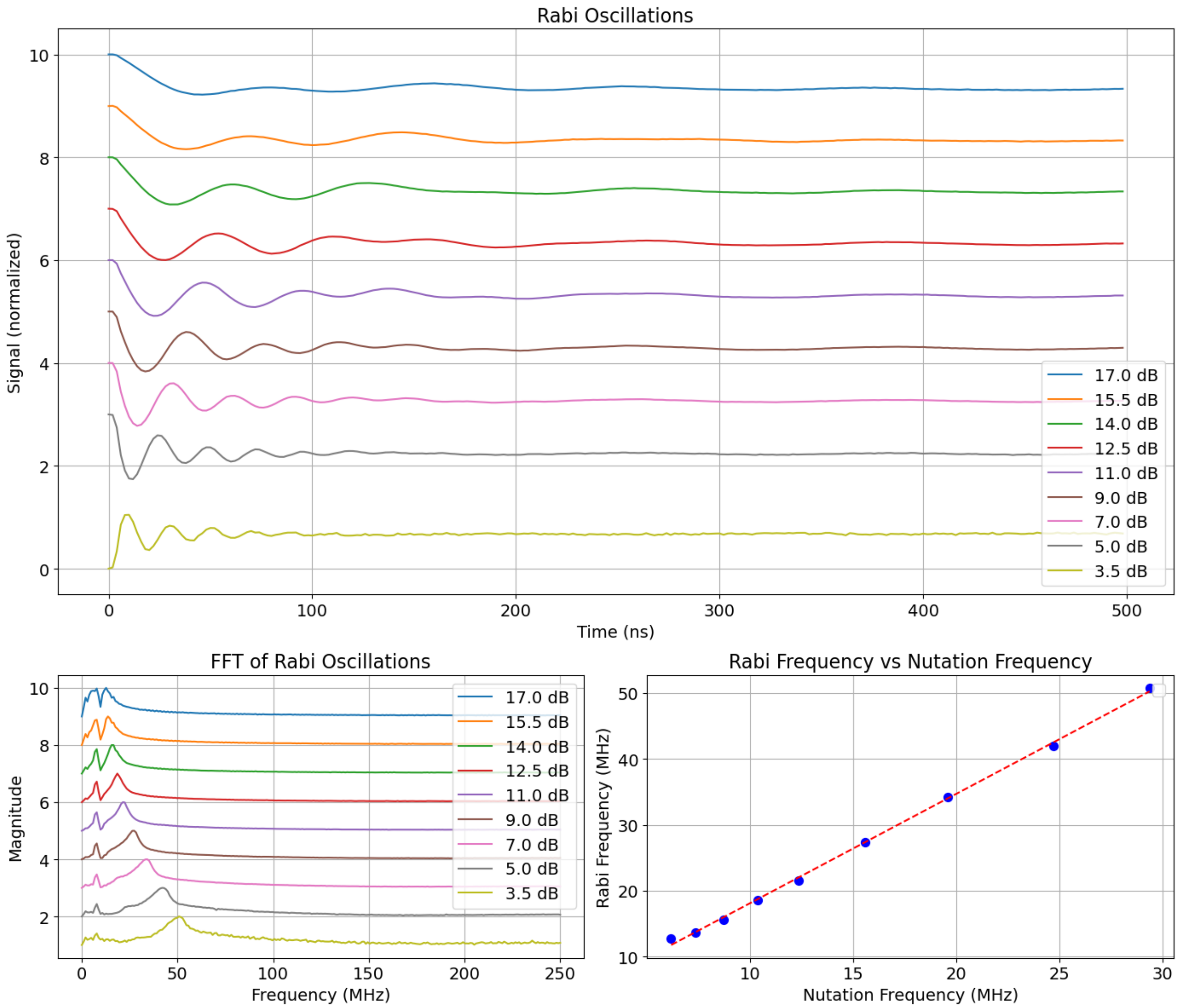

Figure S28. *(Top)* Rabi oscillations for compound **4** measured at 4 K and a magnetic field of 1828 G under different microwave power attenuation levels. *(Bottom left)* Corresponding Fourier transforms, showing an additional narrow peak attributed to the ^{1}H ESEEM effect. *(Bottom right)* Dependence of the experimental Rabi frequency on the nutation frequency, calculated assuming a nutation frequency of 44 MHz at 0 dB attenuation.

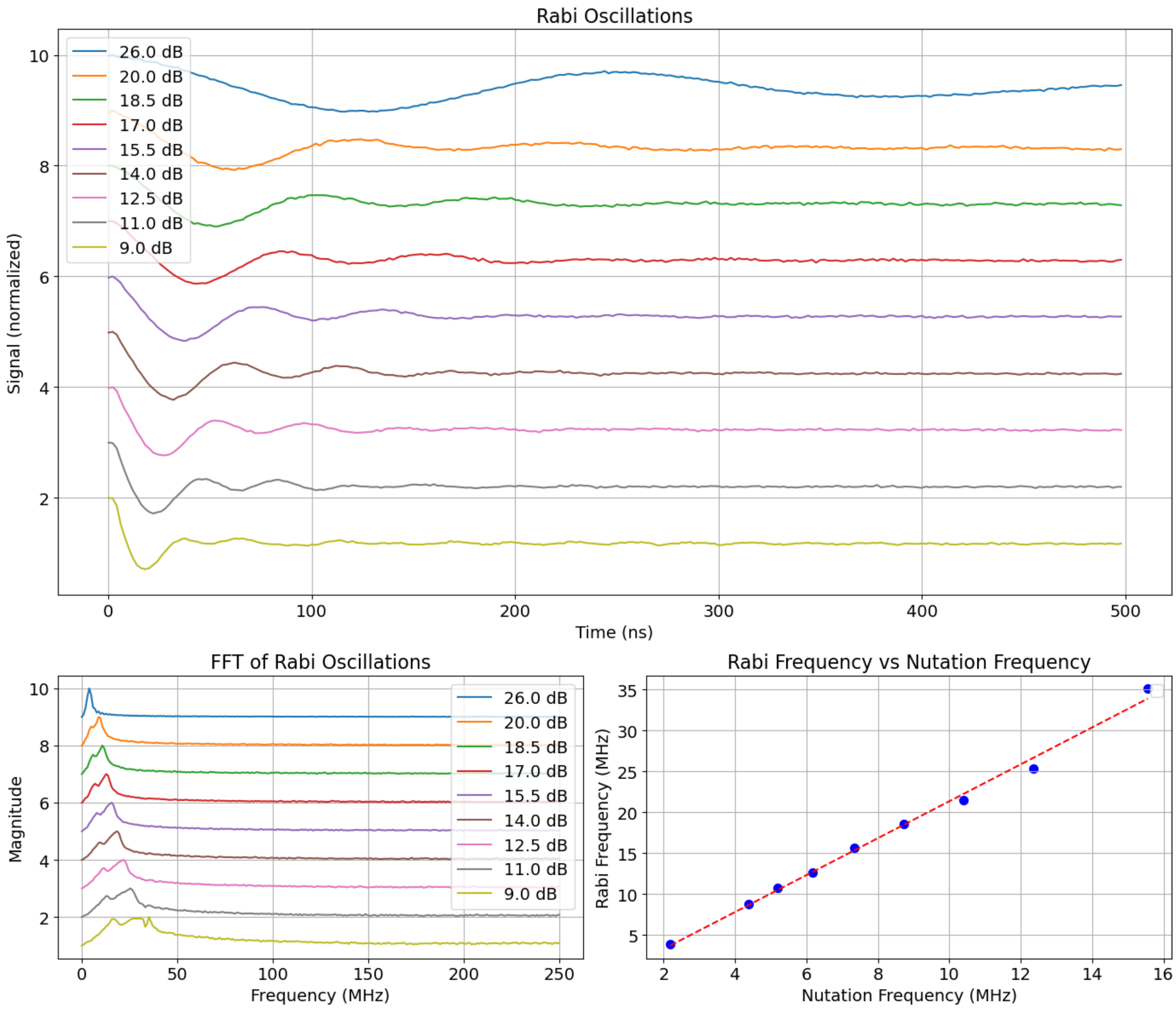

Figure S29. *(Top)* Rabi oscillations for compound **4** measured at 4 K and a magnetic field of 8200 G under different microwave power attenuation levels. *(Bottom left)* Corresponding Fourier transforms, showing an additional narrow peak attributed to the ^{1}H ESEEM effect. *(Bottom right)* Dependence of the experimental Rabi frequency on the nutation frequency, calculated assuming a nutation frequency of 44 MHz at 0 dB attenuation.

7. Tables

Table S1. Crystallographic and refinement parameters for the structures of compounds **1**, **2** and **4**.

Compound	**1**·4.5DMF·4MeOH	**2**·7DMF	**4**·5.5DMF
Formula	$C_{90}H_{72}N_{18}Zn_2$, $C_6O_{12}Cr$, 4.5(C_3H_7NO), 4(CH_4O), Cl	$C_{90}H_{72}N_{18}Zn_2$, $C_6O_{12}Al$, 7(C_3H_7NO), Cl	$C_{90}H_{72}N_{18}Zn_2$, $C_6O_{12}Al_{0.97}Cr_{0.03}$, 5.5($C_3H_7NO$), Cl
FW (g mol^{-1})	2344.97	2374.52	2265.65
Wavelength (Å)	0.72932	0.72932	0.7288
T (K)	100	100	100
Crystal system	monoclinic	monoclinic	monoclinic
Space group	$C2$/c	$C2$/c	$C2$/c
a (Å)	24.040(5)	24.261(5)	24.222(2)
b (Å)	28.327(6)	27.350(6)	26.751(3)
c (Å)	19.235(4)	19.156(4)	19.3424(17)
α (°)	90	90	90
β (°)	117.92(3)	118.99(3)	119.193(3)
γ (°)	90	90	90
V (Å^3)	11574(5)	11118(5)	10941.3(18)
Z	4	4	4
ρ_{calcd} (g cm^{-3})	1.345	1.418	1.375
μ (mm^{-1})	0.684	0.624	0.630
Reflections	5228	7995	13562
R_{int}	0.0214	0.0241	0.0382
Restraints	220	48	55
Parameters	645	639	672
S	1.054	1.060	1.83
R_1 [$I>2\sigma(I)$]	0.1515	0.0795	0.0599
wR_2 [$I>2\sigma(I)$]	0.4380	0.2376	0.1932
R_1 [all data]	0.1665	0.0795	0.0713
wR_2 [all data]	0.4629	0.2491	0.2059
Largest peak / hole (e Å^3)	1.204 / –0.692	0.944 / –0.787	1.281 / –0.711

Table S2. Hydrogen bonds in the supramolecular $M(ox)_3@[Zn_2(L2)_3]$ assemblies in the structures of compounds **1**, **2** and **4**.

	D–H···A	D–H (Å)	H···A (Å)	D···A (Å)	D–H···A (º)
**1**	N3–H3A···O5	0.88	2.11	2.916(17)	152.5
	N4–H4B···O2	0.88	2.28	3.072(17)	149.2
	N9–H9A···O1	0.88	2.05	2.896(14)	161.3
**2**	N3–H3A···O2[a]	0.88	2.27	3.091(6)	156.1
	N4–H4B···O5[a]	0.88	2.13	2.934(6)	151.1
	N9–H9A···O1	0.88	2.11	2.952(5)	158.9
**4**	N3–H3A···O5	0.88	2.13	2.937(3)	152.1
	N4–H4B···O1[b]	0.88	2.21	3.045(3)	157.6
	N9–H9A···O2[b]	0.88	2.11	2.944(2)	158.6

a: 1-x, y, 0.5-z; b: 1-x, y, 1.5-z

Table S3. Metal-donor bond distances (Å) and angles (º) describing the coordination environment of the metal ions in the structures of compounds **1**, **2** and **4**. In the latter, distances and angles are not given for the Cr1 site due to its very low occupancy (*).

Compound	**1**·4.5DMF·4MeOH M = Cr	**2**·7DMF M = Al	**4**·5.5DMF M = Al / Cr*
Zn1–N2	2.133(13)	2.115(5)	2.1229(19)
Zn1–N8	2.145(10)	2.117(4)	2.1032(18)
Zn1–N5[a]	2.153(15)	2.132(4)	2.114(2)
Zn1–N7	2.182(11)	2.205(4)	2.1993(19)
Zn1–N6[a]	2.187(12)	2.169(5)	2.176(2)
Zn1–N1	2.244(15)	2.189(4)	2.176(2)
N2–Zn1–N8	99.6(4)	98.86(17)	99.16(7)
N2–Zn1–N5[a]	95.6(5)	95.40(16)	95.70(7)
N8–Zn1–N5[a]	99.0(5)	99.00(15)	98.04(8)
N2–Zn1–N7	167.8(6)	96.16(17)	168.08(8)
N8–Zn1–N7	76.6(4)	76.29(15)	76.59(7)
N5[a]–Zn1–N7	96.4(5)	168.07(18)	95.93(8)
N2–Zn1–N6[a]	93.4(4)	165.39(17)	96.49(7)
N8–Zn1–N6[a]	166.6(4)	94.47(17)	163.89(7)
N5[a]–Zn1–N6[a]	76.5(7)	76.53(18)	76.56(9)
N7–Zn1–N6[a]	91.2(4)	92.77(19)	88.77(7)
N2–Zn1–N1	76.6(6)	76.52(19)	76.36(8)
N8–Zn1–N1	92.0(5)	164.79(16)	94.64(8)
N5[a]–Zn1–N1	167.5(5)	95.87(16)	166.00(8)
N7–Zn1–N1	91.9(6)	89.69(15)	92.76(8)
N6[a]–Zn1–N1	94.0(6)	92.0(2)	92.71(9)
M1–O2	1.943(10)	1.879(3)	1.8994(18)
M1–O5	1.991(9)	1.891(4)	1.8771(18)
M1–O1	2.001(9)	1.878(4)	1.8896(15)
O2–M1–O5	92.4(4)	93.74(15)	175.79(8)
O2–M1–O5[a]	92.5(4)	91.01(15)	91.79(7)
O2–M1–O1	91.5(4)	85.89(15)	85.32(7)
O2–M1–O1[a]	83.8(4)	89.65(15)	89.45(7)
O5–M1–O1	93.4(4)	91.34(15)	91.63(7)
O5–M1–O1[a]	174.9(4)	175.05(16)	93.84(7)
O1–M1–O1[a]	90.2(5)	92.5(2)	172.55(12)
O2–M1–O2[a]	173.4(6)	173.6(2)	91.09(11)
O5–M1–O5[a]	83.3(6)	85.0(2)	85.50(11)

a: 1-x, y, 1.5-z for **1** and **4**, 1-x, y, 0.5-z for **2**

Table S4. Characteristic relaxation times τ (s) extracted from the frequency dependence of the ac magnetic susceptibility of **1**. Errors are estimates based on the fit statistical error.

B (T)	*T* (K)	τ (s)	τ_{slow} (s)
0.05	2	0.000085(30)	-
0.1	2	0.000138(40)	-
0.2	2	0.000186(60)	-
0.3	2	0.000154(50)	0.183(60)
0.5	2	0.000049(20)	0.195(60)
1	2	0.000039(20)	0.261(80)
0.2	2	0.000198(30)	-
0.2	2.2	0.000158(25)	-
0.2	2.4	0.000129(25)	-
0.2	2.6	0.000113(20)	-
0.2	2.8	0.000096(20)	-
0.2	3	0.000086(20)	-
0.2	3.5	0.000061(20)	-
0.2	4	0.000051(20)	-
0.2	4.5	0.000039(15)	-
0.2	5	0.000033(15)	-
0.2	6	0.000024(15)	-
0.2	7	0.000016(10)	-
